# Scalable synthesis of coordinatively unsaturated metal-nitrogen sites for large-scale CO_2_ electrolysis

**DOI:** 10.1038/s41467-023-36688-6

**Published:** 2023-03-23

**Authors:** Ji Wei Sun, Xuefeng Wu, Peng Fei Liu, Jiacheng Chen, Yuanwei Liu, Zhen Xin Lou, Jia Yue Zhao, Hai Yang Yuan, Aiping Chen, Xue Lu Wang, Minghui Zhu, Sheng Dai, Hua Gui Yang

**Affiliations:** 1 Key Laboratory for Ultrafine Materials of Ministry of Education, Shanghai Engineering Research Center of Hierarchical Nanomaterials, School of Materials Science and Engineering, East China University of Science and Technology, 130 Meilong Road, Shanghai, 200237 China; 2 State Key Laboratory of Chemical Engineering, School of Chemical Engineering, East China University of Science and Technology, 130 Meilong Road, Shanghai, 200237 China; 3 Physics Department and Shanghai Key Laboratory of Magnetic Resonance, School of Physics and Electronic Science, East China Normal University, 3663 North Zhongshan Road, Shanghai, 200062 China; 4 Key Laboratory for Advanced Materials and Feringa Nobel Prize Scientist Joint Research Center, Institute of Fine Chemicals, School of Chemistry and Molecular Engineering, East China University of Science and Technology, 130 Meilong Road, Shanghai, 200237 China

**Keywords:** Carbon capture and storage, Electrocatalysis

## Abstract

Practical electrochemical CO_2_-to-CO conversion requires a non-precious catalyst to react at high selectivity and high rate. Atomically dispersed, coordinatively unsaturated metal-nitrogen sites have shown great performance in CO_2_ electroreduction; however, their controllable and large-scale fabrication still remains a challenge. Herein, we report a general method to fabricate coordinatively unsaturated metal-nitrogen sites doped within carbon nanotubes, among which cobalt single-atom catalysts can mediate efficient CO_2_-to-CO formation in a membrane flow configuration, achieving a current density of 200 mA cm^−2^ with CO selectivity of 95.4% and high full-cell energy efficiency of 54.1%, outperforming most of CO_2_-to-CO conversion electrolyzers. By expanding the cell area to 100 cm^2^, this catalyst sustains a high-current electrolysis at 10 A with 86.8% CO selectivity and the single-pass conversion can reach 40.4% at a high CO_2_ flow rate of 150 sccm. This fabrication method can be scaled up with negligible decay in CO_2_-to-CO activity. In situ spectroscopy and theoretical results reveal the crucial role of coordinatively unsaturated metal-nitrogen sites, which facilitate CO_2_ adsorption and key *COOH intermediate formation.

## Abstract (web summary)

Scalable fabrication of coordinatively unsaturated metal-nitrogen is challenging. Here the authors report a general method for synthesize such material for CO2 electrochemical conversion at a high catalytic current of 10 A for more than 60 h stability using a 100 cm2 membrane flow cell.

## Introduction

Electrochemical CO_2_ reduction reaction (CO_2_RR) provides a sustainable way to produce value-added chemicals and fuels when combined with renewable electricity^1–4^. Among the main products of CO_2_RR^5–7^, CO is one of the most likely products to achieve high economic returns not only because of its high selectivity and activity, but also for its facile separation in liquid water for further application, such as syngas in bulk chemicals manufacturing and tandem reaction for electrochemical CO_2_-to-multicarbons conversion^8–12^. However, the low CO_2_ conversion efficiency makes it difficult to achieve high economic returns^13,14^. The single-pass conversion (SPC) of CO_2_ is closely related to the cost of CO_2_RR feed and product separation. Higher SPC means that more CO_2_ can be converted. For the product separation cost, taking the conversion of CO_2_ to CO as an example, when the CO_2_ SPC is 10%, the cost of separating gas products by pressure swing adsorption accounts for about 23% of the total cost. However, when the CO_2_ SPC achieves 50%, this value will drop to 6%^15^. In alkaline or neutral electrolyte, two OH^-^ will be produced for each generation of CO, so that an additional CO_2_ will be consumed to generate carbonate. Therefore, in theory, SPC will not exceed 50%, especially under alkaline conditions. Although this problem can be solved under acidic conditions, it is often accompanied by serious hydrogen evolution reactions. Recently, Edward H. Sargent et al. reported the realization of more than 50% SPC under acidic conditions, which is a major breakthrough in the field^16^. However, it can only be realized at a low CO_2_ flow rate (3 sccm), so it is still a great challenge for real industrial applications. Therefore, according to the definition of SPC (formula 1), realizing high CO selectivity (FE_CO_) and large operating current (I) for CO_2_-CO conversion at a high CO_2_ flow rate is a very critical scientific issue.

1 $${{{{{{\mathrm{SPC}}}}}}}=\frac{{{{{{\mathrm{C}}}}}}{{{{{{\mathrm{O}}}}}}}_{2}\,{{{{{{\mathrm{consumption}}}}}}}}{{{{{{{{\mathrm{CO}}}}}}}}_{2}\,{{{{{{\mathrm{inflow}}}}}}}}\propto \frac{I\times {{{{{{{\mathrm{FE}}}}}}}}_{{{{{{{\mathrm{CO}}}}}}}}}{{{{{{{{\mathrm{CO}}}}}}}}_{2}\,{{{{{{\mathrm{flow}}}}}}}\,{{{{{{\mathrm{rate}}}}}}}}$$

Recently, atomically dispersed metal-nitrogen active sites embedded in carbon-based support (M-N-C) provide an intriguing paradigm for CO_2_-to-CO conversion due to their high atom utilization efficiency, unique electronic structures, and impressive Faradaic efficiency (FE), showing a huge potential to replace the precious metal-based CO_2_RR catalysts^17–21^. However, these M-N-C catalysts were usually fabricated via high-temperature treatment (e.g., 800 °C for several hours), which would induce energy consumption and metal species aggregation; even worse, their local fine structure modification (e.g., valent states and coordination environments) aiming to maintain considerable selectivity and activity, especially at gram-scale fabrication, still remain challenges in this field. Although these M-N-C catalysts have excellent CO selectivity in the H-type cell or flow cell, the comprehensive CO_2_ reduction performance (CO selectivity, the energy efficiency of the cell, and stability, etc.) still needs further investigation when high-current electrolysis of CO_2_ is performed in an anion membrane electrode assembly (MEA). In this regard, large-area devices also show urgent requirements for the large-scale preparation of high-activity catalysts. With this in mind, we anticipate controllable and economically viable fabrication of M-N-C catalysts, which feature active local structures and can be scaled-up, will be a breakthrough in achieving high SPC in CO_2_-CO electrolysis with high current and high selectivity.

Herein, we report a microwave-assisted strategy to fabricate coordinatively unsaturated metal-nitrogen sites doped within defective carbon nanotubes (Fe, Co, or Ni-CNTs-MW) as efficient CO_2_-to-CO electrocatalysts. This simple synthetic approach is universal for gram-scale production within ~2 min fabrication, showing negligible mass loss during the process. As a proof-of-concept nonprecious catalyst, Co-CNTs-MW delivers a high-efficiency CO_2_-to-CO conversion in an H-type cell, exhibiting over 90% FE_CO_ in the wide potential range of −0.60 to −1.00 V (all potentials were referenced to reversible hydrogen electrode, vs. RHE, and not *iR*-corrected) in 0.5 M KHCO_3_ aqueous electrolyte. The Co-CNTs-MW catalyst can deliver industrial-current CO_2_ electrolysis in the flow cell configuration, with FE_CO_ of 96.0% at the current density (*j*) up to 350 mA cm^−2^. Moreover, this catalyst sustains high-rate CO_2_-to-CO formation in MEA, achieving a *j* of 200 mA cm^−2^ with FE_CO_ of 95.4% and FCEE of 54.1% (two-electrode cell voltage of 2.34 V, all cell voltages were not *iR*-corrected), which outperforms most nonprecious catalysts-based electrolyzers. In the MEA sized of 100 cm^2^, the catalyst demonstrates a selective amperage-current CO_2_ electrolysis up to 10 A with FE_CO_ of 86.8%, and SPC can reach 40.4% at a high CO_2_ flow rate of 150 sccm. The long-term test of this electrolyzer was stable at 2 A for more than 60 h. Our in situ attenuated total reflectance infrared spectroscopy (ATR-IR) and density functional theory (DFT) calculations ambiguously prove that atomically dispersed coordinatively unsaturated Co-N sites not only activate CO_2_ adsorption but also facilitate the *COOH formation and inhibit the occurrence of HER, thus accelerating the overall CO_2_-to-CO conversion.

## Results

### Local structural characterization of Co-CNTs-MW

**Fig. 1:**
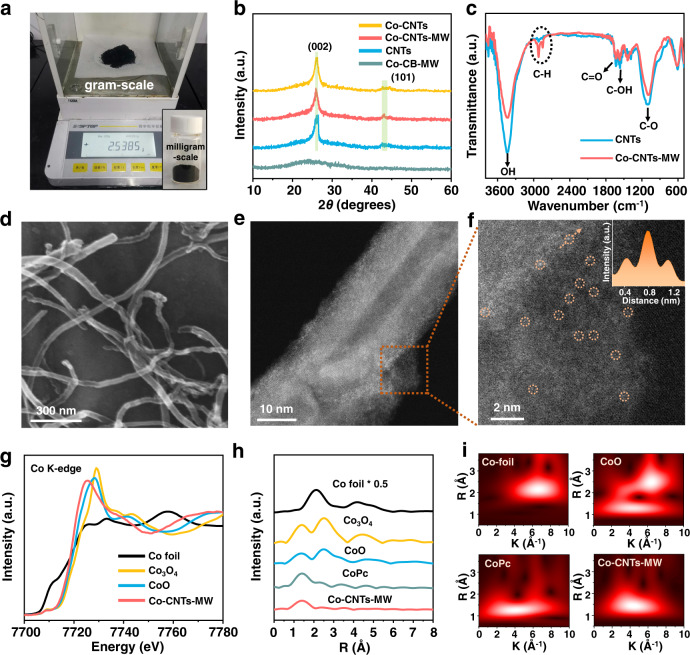
Structural and morphology characterization of Co-CNTs-MW. **a** Digital photograph of the synthesized Co-CNTs-MW at milligram-scale (insert) and a gram-scale. **b** XRD patterns of Co-CNTs, Co-CNTs-MW, CNTs, and Co-CB-MW, respectively, showing no obvious diffraction peaks of crystalline metal or metal oxide species. **c** FTIR spectra of pristine CNTs and Co-CNTs-NW, suggesting that the peak intensities of oxygen-containing groups were obviously decreased after microwave treatment. **d** SEM image of Co-CNTs-MW, showing typical morphology of CNTs. **e**, **f** Aberration-corrected HAADF-STEM image of Co-CNTs-MW at different magnifications. The atomically dispersed Co sites were marked by orange circles and the corresponding intensity profiles (insert) along the orange line in **f**. **g** Co K-edge XANES spectra of Co-CNTs-MW and reference Co foil, Co_3_O_4_, and CoO samples, which shows the oxidized Co^δ+^ in Co-CNTs-MW. **h** Co K-edge Fourier transformed EXAFS spectra in the R space of Co-CNTs-MW and reference Co foil, Co_3_O_4_, CoO, and CoPc samples. **i** WT-EXAFS plots of Co foil, CoO, CoPc, and Co-CNTs-MW samples. The EXAFS and WT-EXAFS data demonstrate the isolated coordinatively unsaturated Co-N sites in Co-CNTs-MW.

Taking advantage of the carbon material’s ability to absorb microwave and heat rapidly, we have prepared M-N-C catalysts with precise structure^22,23^. Figure S1 shows the schematic illustration of rapid synthesis of typical Co-CNTs-MW prepared by microwave. This method shows universality and scalability (gram-level preparation) (Fig. 1a), which would be extended for the preparation of single-atom catalysts with fine structure. The experimental details and general synthesis protocols were referred to in the Supplementary Information (experimental section and Fig. S2). To be specific, CNTs were firstly hydroxylated with functional groups to anchor Co complex precursor. The molecular complex of 1,10-phenanthroline (1,10-phen) was chosen to coordinate Co ions^24^, which were then adsorbed onto CNTs to form Co-CNTs for further microwave treatment. During the microwave heating process, CNTs would absorb the microwave and create a local high-temperature environment, which could induce carbon loss to form defects or vacancies via generating CO or CO_2_ because of the oxygen-containing functional groups. At the same time, the local high-temperature environment could not only introduce N heteroatoms from 1,10-phen to form N-doped CNTs but also induce Co atoms to heat transfer onto the defective sites on CNTs, thus fabricating coordinatively unsaturated Co-N sites. The resultant Co-CNTs-MW samples were fabricated at milligram-scale (~140 mg), and could also be once scaled-up to gram-scale (~2.5 g), shown in Fig. 1a. In order to compare the effects of different microwave absorbents, carbon black was also used to synthesize Co-CB-MW samples. Compared with the traditional heat treatment via tube furnace equipment, this method shows potential advantages of short heating time (~2 min), less energy consumption, selective heating (Fig. S3), less metal agglomeration (Fig. S4), and almost no mass loss (Fig. S5). Details of the above advantages were proved and described in the Supplementary Information.

Two diffraction peaks at 26.5° and 44.6° were shown in the X-ray diffraction (XRD) patterns of CNTs, Co-CNTs, and Co-CNTs-MW (Fig. 1b), ascribing to the characteristic peaks of the (002) and (101) crystal planes of graphite carbon^25^. Notably, no diffraction peaks of metallic Co or Co oxides were observed, implying Co species were highly dispersed over the CNTs surfaces. In the Fourier transform infrared spectroscopy (FTIR) spectra (Fig. 1c), the Co-CNTs-MW catalyst obtained by microwave treatment shows obviously decreased intensities of oxygen-containing groups than those of pristine CNTs. This indicates that oxygen-containing groups and carbon atoms might be lost in the form of CO or CO_2_ in a high-temperature environment, which provides defective sites for anchoring Co-N sites. Scanning electron microscopy (SEM) images (Fig. 1d and Fig. S6) show that the morphology of CNTs was not destroyed after microwave irradiation. In addition, aberration-corrected scanning transmission electron microscopy (AC-STEM) was utilized to visualize the isolated Co sites over the CNTs (Fig. 1e, f). In the high-angle annular dark-field-STEM (HAADF-STEM) image (Fig. 1f), atomically dispersed Co sites are identified as isolated high Z-contrast spots, as highlighted by the dotted orange circles. The corresponding intensity profile along the orange line (inset of Fig. 1f) further demonstrates that this is the point. Energy-dispersive X-ray spectroscopy (EDS) elemental maps (Figure S7) of Co-CNTs-MW further illustrate the overlapped distribution of N and Co on CNTs without obvious aggregation. The mass loading of Co was determined to be 0.18 wt% by inductively coupled plasma mass spectrometry (ICP-MS).

The Raman spectra display that the value of I_D_/I_G_ decreased after microwave heating (from 0.76 to 0.61 after microwave treatment for Co-CNTs), which indicates an increased graphitization degree at a high pyrolysis temperature (Fig. S8)^26^. The valence states and chemical compositions of Co-CNTs-MW were investigated by X-ray photoelectron spectroscopy (XPS). The XPS survey spectra show that only C, N, O, and Co elements exist in Co-CNTs-MW. The N 1 *s* spectrum of Co-CNTs-MW was deconvoluted into pyridine N (398.2 eV), Co-N (399.4 eV), and graphitic N (401.4 eV) peaks, respectively^27^. The Co 2*p* spectrum shows the peaks located at 781.1 and 797.4 eV are assigned to oxidized Co species in the Co 2*p*_3/2_ and Co 2*p*_1/2_ regions, respectively (Fig. S9)^28^. The Brunauer-Emmett-Teller (BET) surface areas of CNTs, Co-CNTs, and Co-CNTs-MW were obtained of 123.99, 160.09, and 113.31 m^2^ g^−1^, respectively (Fig. S10). The difference may be attributed to that the irregular surface of the decorated molecular complex. For comparison, the specific surface area decreases after microwave heating, which indicates that Co atoms complexed by 1,10-phen were successfully integrated into CNTs.

For further information regarding the electronic structure and local coordination environment of Co atoms in Co-CNTs-MW, X-ray absorption near-edge structure (XANES) and extended X-ray absorption fine structure (EXAFS) analyses were carried out. The Co K-edge XANES profiles in Fig. 1g indicate that Co species in Co-CNTs-MW are in a higher oxidation state than that of Co foil but lower than that of CoO, demonstrating the distinctive electronic structure of Co^δ+^ (0 <  δ  < 2)^29^, which is consistent with the XPS results. In the EXAFS spectra (Fig. 1h and Fig. S11), Co foil exhibits a typical first shell Co-Co pair at 2.20 Å (all bond lengths were not phase-corrected). Co-CNTs-MW displays one main peak at 1.45 Å, while no Co-Co bond was detected, indicating the existence of atomically dispersed Co-N sites. The peak area of the Co-N bond is positively correlated with the coordination number^30,31^. The area of the Co-N peak of Co-CNTs-MW is obviously lower than that of CoPc (Co-N_4_), indicating the lower coordination structures of Co-N_x_ configurations in the microwave-derived samples (x < 4). The wavelet transforms (WT) results^32^ provide further support for the existence of a Co-N bond (with a maximum at 3.4 Å^−1^) in the Co-CNTs-MW catalyst, as compared with Co foil, CoO, and CoPc references (Fig. 1i). The exact coordination configuration of Co atom can be obtained by EXAFS fitting (Fig. S12a and Table S1)^33^. The best-fitting analysis clearly confirms that the Co-N coordination number is ~3.0 and the Co-O coordination number is ~2.1. By fitting the EXAFS of Co-CNTs-MW after CO_2_RR, it was found that the oxygen coordination number decreased significantly (from 2.1 to 0.9), while the nitrogen coordination number almost did not change, indicating that the oxygen coordination structure was extremely unstable and would gradually disappear with the reaction. In addition, XPS spectra in the O 1 *s* region and STEM-based electron energy loss spectroscopy (EELS, Fig. S12b, c) of Co-CNTs-MW also show that oxygen in the catalyst mainly exists in the form of unstable adsorbed oxygen. Therefore, the X-ray absorption fine structure (XAFS) results unambiguously elucidate the existence of characteristic coordinatively unsaturated Co-N_3_ sites in Co-CNTs-MW.

### Electrocatalytic CO_2_-to-CO performance evaluation

**Fig. 2:**
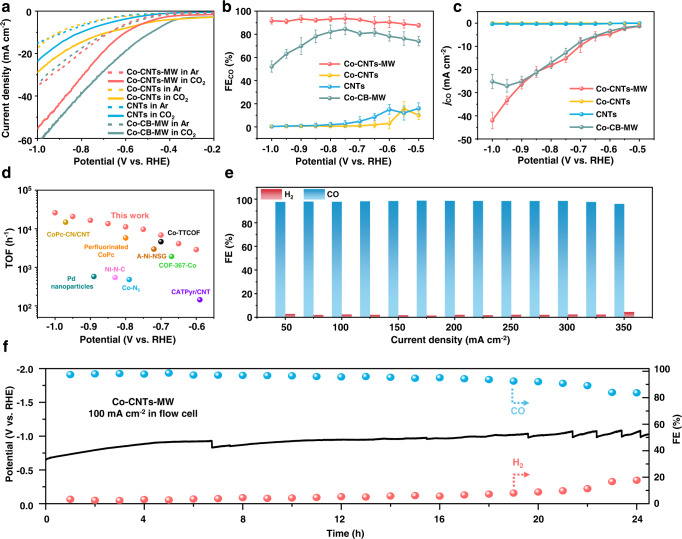
Evaluation of CO_2_RR performances in the H-type cell and flow cell. **a** LSV curves acquired in CO_2_-saturated (solid line) or Ar-saturated (dashed line) 0.5 M KHCO_3_ electrolyte, **b** CO Faradaic efficiencies and **c** CO partial current densities at various applied potentials on Co-CNTs-MW, Co-CNTs, CNTs, and Co-CB-MW in the H-type cell, respectively. The error bars represent the standard deviation of three measurements. **d** Calculated TOF of Co-CNTs-MW catalyst from **c** compared with state-of-the-art CO_2_-to-CO conversion electrocatalysts. A-Ni-NSG^19^, Co-N_5_^35^, CoPc-CN/CNT^36^, COF-367-Co^37^, Perfluorinated CoPc^38^, Pd nanoparticle^39^, Co-TTCOF^40^, CATPyr/CNT^41^, and Ni-N-C^42^ were selected as references. More details were summarized in Table S2. **e** CO and H_2_ Faradaic efficiencies on Co-CNTs-MW catalyst in the range of current densities from 50 to 350 mA cm^−2^ in a flow cell configuration with 1.0 M KOH as electrolyte. **f** Long-term electrolysis under a current density of 100 mA cm^−2^ in the flow cell. After 20 h of testing at a current density of 100 mA cm^−2^, the catalyst still has a CO selectivity of up to 92.1%. The loadings of catalysts are 1.0 mg cm^−2^.

The electrocatalytic properties of Co-CNTs-MW for CO_2_RR were first evaluated in a standard three-electrode H-type cell configuration using CO_2_-saturated 0.5 M KHCO_3_ as the electrolyte (more details were shown in Supplementary Information). Linear sweep voltammetry (LSV) measurements for Co-CNTs-MW and control samples were conducted in Ar- and CO_2_-saturated 0.5 M KHCO_3_ solution, respectively (Fig. 2a). The Co-CNTs-MW catalyst exhibits a much higher current density in CO_2_-saturated KHCO_3_ solution than that in Ar-saturated solution, indicating that the currents measured under CO_2_ saturation are evidently contributed from the electrocatalytic CO_2_ reduction. All the control samples show the same tendency in the *j* difference under CO_2_ and Ar atmosphere. The *j* of Co-CNTs-MW is slightly lower than that of Co-CB-MW, which may be attributed to the better conductivity and larger BET surface areas of carbon black (Fig. S10)^34^. The gas phase products under bulk electrolysis were quantified by online gas chromatograph. In CO_2_-saturated 0.5 M KHCO_3_, Co-CNTs-MW exhibits higher CO Faradaic efficiency than those of CNTs and Co-CNTs controls (Fig. 2b). A high plateau of FE_CO_ over 90% was retained under a broad potential range from −0.60 to −1.00 V, with a maximum CO selectivity of above 93.6% FE at −0.75 V (vs. RHE, all potentials were not *iR*-corrected in this work). The CO selectivity of Co-CB-MW is lower than that of Co-CNTs-MW. This is caused by the poor microwave absorption performance of carbon black^23^, resulting in the coordinatively unsaturated Co-N sites could not be well anchored on the carbon black substrate (Fig. S13). As shown in Fig. 2c, Co-CNTs-MW exhibits a partial current density (*j*_CO_) of 42 mA cm^−2^ at −1.00 V, about 130 times larger than that of CNTs (0.32 mA cm^−2^). As a comparison, the Co-CNTs-800 °C catalyst was prepared by calcination in a tubular furnace at 800 °C. Compared with Co-CNTs-MW, the CO selectivity and stability of Co-CNTs-800 °C decreased significantly (Fig. S14), which may be due to the Co-CNTs-800 °C containing more metal agglomeration (Fig. S4). In addition, the Co-CNTs-MW catalyst displays a high turnover frequency (TOF) value of 25896 h^−1^ for CO_2_RR at −1.00 V, which is much higher than that of the state-of-the-art heterogeneous electrocatalysts under similar conditions (Fig. 2d and Table S2)^19,35–42^. The calculated electrochemical active specific area (ECSA) of Co-CNTs-MW, based on double-layer capacity, shows no obvious changes compared with those of CNTs, Co-CNTs, and Co-CB-MW (Fig. S15a–e), which further demonstrates the intrinsic activity of coordinatively unsaturated Co-N sites. Electrochemical impedance spectroscopy (EIS) was carried out to gain further insight into CO_2_RR kinetics. The Nyquist plot of Co-CNTs-MW shows much smaller interfacial charge-transfer resistance during the CO_2_ reduction process compared with CNTs and Co-CNTs controls (Fig. S15f), suggesting a favorable Faradaic process^43^. After replacing CO_2_ with Ar gas, the reduction products become H_2_, which demonstrates that the carbon in CO comes from CO_2_ instead of impurities (Fig. S16).

In order to overcome the CO_2_ mass-transport issue in batch-type electrolysis, we further used the flow cell configuration which can construct a gas-liquid-solid three-phase interface for CO_2_RR and avoid the problem of low diffusion and solubility in the aqueous solution (Fig. S17). As a result, Co-CNTs-MW shows FE_CO_ above 96% in a wide range of current density from 50 to 350 mA cm^−2^ (Fig. 2e). This high selectivity in the flow cell configuration indicates a huge potential of gas-diffusion electrolysis in the real-world full-cell electrolysis. For the long-term electrolysis under a current density of 100 mA cm^−2^ in the flow cell, the catalyst still shows a CO selectivity of up to 92.1% after 20 h (Fig. 2f). After long-term electrolysis, GDE was broken down, obvious water droplets appeared on the back (Fig. S18) and the electrochemical curve also fluctuated significantly. This problem can be solved under the test system of MEA.

### Structural stability of Co-CNTs-MW

To investigate the structure of the catalyst after reaction, we carried out a series of characterizations of the Co-CNTs-MW catalyst after reacting for one hour at the current density of 100 mA cm^−2^ in the flow cell. As shown in Fig. S19a, no diffraction peak of metallic Co or Co oxide was observed in the XRD patterns of Co-CNTs-MW after CO_2_RR. In the EXAFS spectra (Fig. S19b), Co-CNTs-MW after CO_2_RR did not show the peak of metallic Co at 2.20 Å (all bond lengths were not phase-corrected), which indicates that the Co-CNTs-MW catalyst did not induce metal agglomeration after CO_2_RR. EXAFS fitting result (Fig. S12a and Table S1) shows that the coordination structure of nitrogen has almost no change before and after CO_2_RR (from 3.0 to 3.3), and it is still the unsaturated coordination structure of Co-N_3_. XPS characterization was used to test whether the valence state of Co in the catalyst changed after the reaction. The Co 2*p* spectrum of Co-CNTs-MW after CO_2_RR (Fig. S19c) shows that the peaks located at 781.2 and 797.3 eV are assigned to oxidized Co species in the Co 2*p*_3/2_ and Co 2*p*_1/2_ regions, which is similar to the Co 2*p* spectrum of Co-CNTs-MW (781.1 and 797.4 eV) (Fig. S19d). This indicates that the valence state of Co-CNTs-MW shows no obvious changes after the reaction. Moreover, in the aberration-corrected HAADF-STEM image (Fig. S19e, f), atomically dispersed Co sites are identified as isolated high Z-contrast spots and highlighted with dotted orange circles. The above characterization tests have shown that the structure of the catalyst has not significantly changed after the reaction, which demonstrates that the catalyst is relatively stable.

### Performance verification in a two-electrode MEA with high FCEEs

**Fig. 3:**
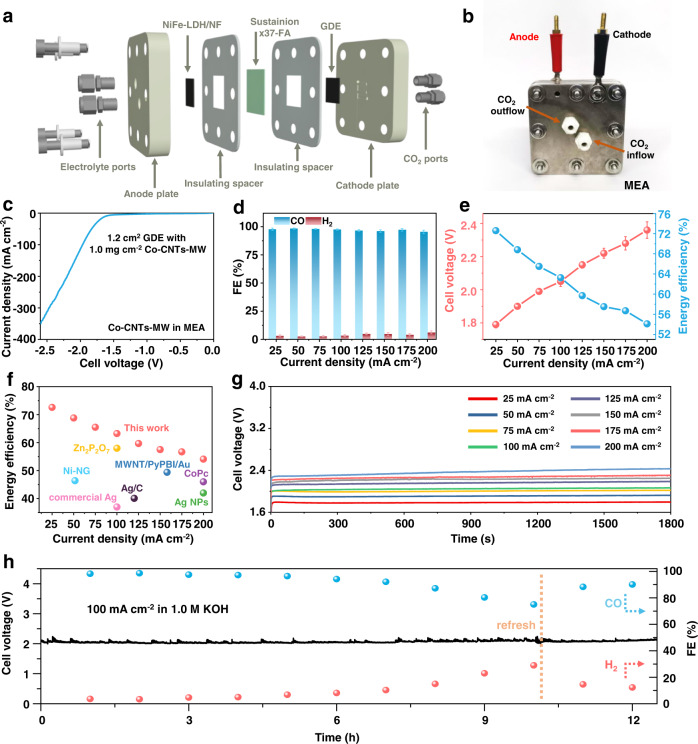
Evaluation of CO_2_RR performances in MEA full-cell configuration. **a** 3D structural diagram and **b** photograph of MEA configuration. **c** LSV curve of Co-CNTs-MW in MEA at a scan rate of 10 mV s^−1^. **d** CO and H_2_ Faradaic efficiencies on Co-CNTs-MW catalyst in the range of current densities from 25 to 200 mA cm^−2^ in MEA. **e** Cell voltage and full-cell energy efficiency on Co-CNTs-MW catalyst in MEA (all cell voltages were not *iR*-corrected). The error bars represent the standard deviation of three measurements. **f** Full-cell energy efficiency of Co-CNTs-MW catalyst compared with state-of-the-art CO_2_-to-CO conversion electrocatalysts. CoPc^44^, Ni-NG^18^, MWNT/PyPBI/Au^45^, Ag nanoparticles^46^, Ag/C^47^, commercial Ag^48^, and Zn_2_P_2_O_7_^49^ were selected as references. More details were summarized in Table S3. **g** Cell voltage at different current densities in 1800 s electrolysis. **h** Long-term electrolysis under a current density of 100 mA cm^−2^ in MEA. The electrolyte tested in **c**–**h** is 1.0 M KOH. The loading of Co-CNTs-MW is 1.0 mg cm^−2^.

Based on the above excellent performance in the three-electrode system, we evaluated the Co-CNTs-MW catalyst in the full-cell anion MEA configuration (Fig. S20). MEA can prevent the catalyst from directly contacting with water and facilitate CO_2_ mass transport, greatly suppressing the competing HER even under large overpotentials^10^. As shown in Fig. 3a, b, the cathode (Co-CNTs-MW GDE) and anode (NiFe-LDH/NF used in 1.0 M KOH or IrO_2_/Ti felt used in 0.1 M KHCO_3_, and their preparation methods were shown in Supplementary Information) are separated by an anion membrane, and dry CO_2_ and electrolyte flow in and out from the cathode and anode plates, respectively. In the full-cell electrolysis configuration for CO_2_RR, Co-CNTs-MW shows a high *j* of 311 mA cm^−2^ at a cell voltage of 2.5 V (Fig. 3c, all cell voltages were not *iR*-corrected in this work). At the same time, a high plateau of FE_CO_ over 95% was retained under a broad current density range from 25 to 200 mA cm^−2^ in 1.0 M KOH electrolyte (Fig. 3d). The full-cell energy efficiency was recorded considering selectivity and applied voltage energy (Fig. 3e). The FCEEs are significantly higher than those of the catalysts reported at the same current density (Fig. 3f). When the *j* increases to 200 mA cm^−2^, the cell voltage is only 2.34 V, which makes the FCEE as high as 54.1% (Fig. 3f). This FCEE value is significantly higher than the recently reported catalysts^18,44–49^, such as Ag^48^ and CoPc-based MEA electrolyzers^44^ (Table S3). Furthermore, to show the reliability of FCEE in this work, the cell voltages at different current densities were recorded when tested in 1.0 M KOH electrolyte for continuous 1800 s (Fig. 3g), showing stable chrono-potentiometric curves. The representative long-term electrolysis was conducted at 100 mA cm^−2^ (Fig. 3h), which shows the stability of catalytic selectivity. Although the selectivity of CO decreased to 75.0% after 10 h, which is mainly attributed to the excessively high-concentration electrolyte causing serious salt accumulation on the back of the GDE and hindering the reaction (Fig. S21), instead of the catalyst inactivation, this problem can be solved by refreshing the electrolyte or applying periodic reductions^50,51^. After refreshing the GDE with deionized water and replacing the electrolyte, the selectivity of CO was restored to 90.1%, demonstrating the robustness of the catalyst on the GDE. The typical ^1^H NMR spectrum of the Co-CNTs-MW after 10 h electrolysis at 100 mA cm^−2^ suggests no detectable liquid product, and the representative gas product distribution further indicates the main product of CO during CO_2_ electrolysis (Fig. S22 and Table S4).

### Amperage-current CO_2_-to-CO electrolysis with high single-pass conversion

**Fig. 4:**
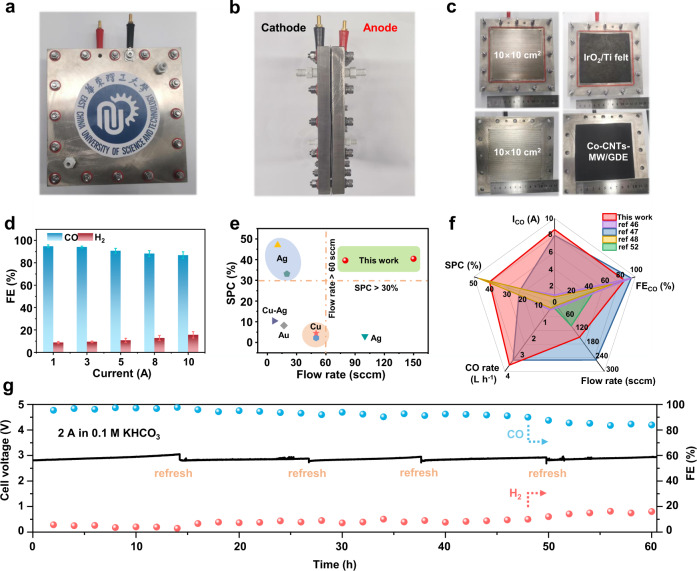
Amperage-current CO_2_-to-CO electrolysis in 100 cm^2^ MEA. **a** Front-viewed, **b** side-viewed, and **c** runner-viewed photograph of the homemade MEA configuration (100 cm^2^). **d** CO and H_2_ Faradaic efficiencies on the Co-CNTs-MW catalyst in the range of current from 1 to 10 A in the MEA sized of 100 cm^2^. The error bars represent the standard deviation of three measurements. **e** The single-pass conversion of Co-CNTs-MW catalyst compared with CO_2_RR electrocatalysts (CO_2_ flow rate: 80 sccm from 1 to 5 A and 150 sccm from 8 to 10 A). More details were summarized in Table S6. **f** Spider chart comparing the performance metrics constructed from the data published for CO_2_-to-CO electrolyzers. **g** Long-term electrolysis under a current of 2 A in MEA sized of 100 cm^2^ (0.1 M KHCO_3_). After 50 h of amperage-current electrolysis under a current of 2 A, the catalyst still behaves FE_CO_ nearly over 90%, and exhibits FE_CO_ of 85.6% after 60 h. The cell voltage keeps around ~2.88 V when excluding the external resistance (~3.10 V without excluding the external resistance) during the electrolysis. The loading of Co-CNTs-MW is 1.0 mg cm^−2^.

Considering the better response to the high stability and high-current requirements of CO_2_RR industrialization, we tried to verify the amperage-current electrolysis in a larger device under the neutral condition. First of all, the CO_2_-to-CO performance of Co-CNTs-MW was evaluated in 0.1 M KHCO_3_ using the MEA size of 1.2 cm^2^, which shows a selective and stable CO_2_ electrolysis process, achieving FE_CO_ of 91.6% at a *j* of 200 mA cm^−2^ (Fig. S23). This indicates that the Co-CNTs-MW catalyst features excellent CO selectivity under neutral conditions in a membrane flow reactor. With this in mind, a homemade MEA sized of 10 × 10 cm^2^ was designed and processed for amperage-current CO_2_ electrolysis under the neutral condition (Fig. 4a–c). At first, 1.0 M KOH was used for testing. Although it still shows excellent CO selectivity at currents of 2 and 5 A, respectively, the cell voltage has increased by 0.3 V in just 500 s, mainly due to the part of the CO_2_ introduced will react with KOH to form carbonate (Fig. S24). In addition, when the current increases to the ampere level, the external resistance of MEA (wire resistance and internal resistance of power supply) cannot be ignored. We first tested that the external resistance of MEA is 0.11 Ω, and all test voltages exclude the influence of external resistance except the stability test (Table S5). As shown in Fig. 4d, the Co-CNTs-MW catalyst can deliver high-selectivity CO_2_ electrolysis with FE_CO_ over 85% at applied constant currents from 1–10 A in 0.1 M KHCO_3_, showing FE_CO_ of 94.8, 94.1, 90.8, 88.2, and 86.8% at currents of 1, 3, 5, 8, and 10 A, respectively. The calculation indicated that with the increase of potential, the number of electron transfers of HER (∆q = 0.85|e|) is greater than that of CO_2_RR (∆q = 0.66|e|), which leads to the gradual enhancement of HER activity (Fig. S25). At a CO_2_ flow rate of 150 sccm, the Co-CNTs-MW has a CO_2_ conversion efficiency of 40.4% at 10 A (the gas outlet flow rate: 102 sccm). Compared with the reported results (Table S6), we have achieved high SPC of CO_2_-to-CO electrolysis with high current, high CO_2_ flow rate, and high CO selectivity (Fig. 4e, f)^46–48,52^. When the current reaches up to 10 A, the production rate of CO is extremely fast, and it would only take ~6.2 h to produce 1 mol CO (3.63 L CO per hour) based on the above amperage-current evaluation (Fig. S26) (the detailed comparison of CO generation rate of different catalysts would be seen in Table S7). In addition, when the current decreases to 1 A, the calculated energy of producing 1 mol CO is the lowest, only 0.15 kW h, which is lower than 0.24 kW h when the current is 10 A (Fig. S26). In order to find a balance between CO generation rate and energy consumption, we selected a current of 2 A for the stability test. In Fig. 4g, our catalyst can continuously drive 2 A-level CO_2_ electrolysis, showing FE_CO_ generally over 90.0% for nearly 50 h, and even after 60 h electrolysis, the CO selectivity remained 85.6%. It is mentionable that the applied cell voltage keeps ~3.10 V (~2.88 V when excluding the external resistance) at 2 A for more than 60 h (Table S8), exhibiting nearly no decay in voltage, which holds great promise for carbon recycling when powered by solar- or wind-generated electricity, even considering the fluctuating behavior of regenerable energy. We highlight that this is the first example of MEA sustaining 2 A-level CO_2_-to-CO electrolysis for more than 50 h. It is believed that this low-cost catalyst possessing high SPC and high stability under high currents would speed up the pace of CO_2_RR towards industrial production.

### In situ ATR-IR analysis and DFT calculations

**Fig. 5:**
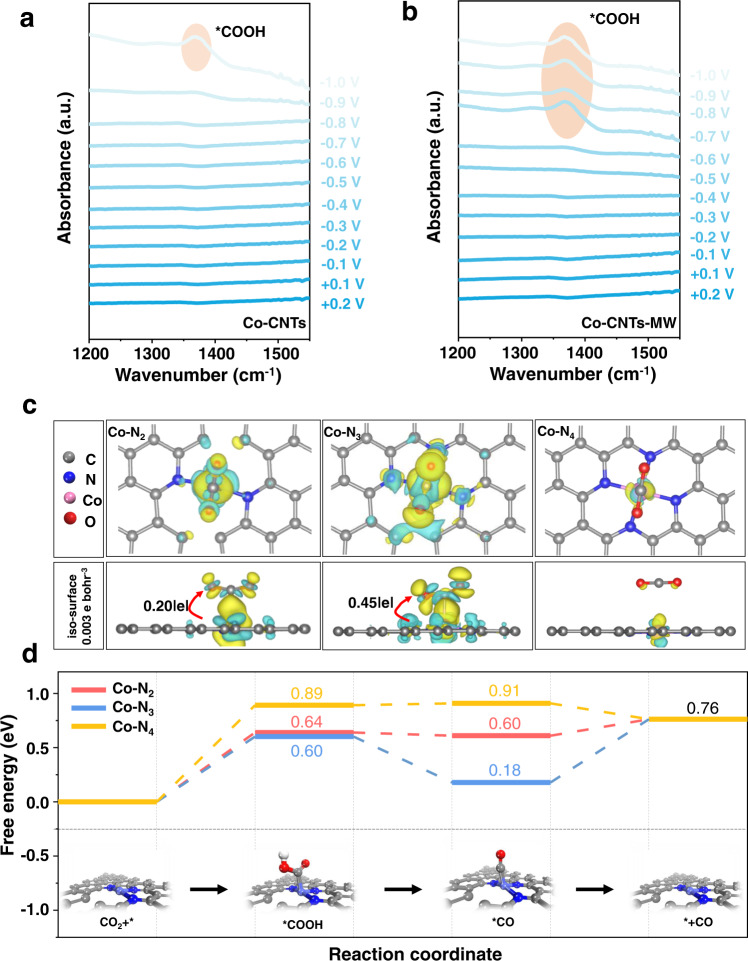
In situ ATR-IR spectra and DFT calculations. **a**, **b** In situ ATR-IR spectra were recorded while ramping down the potential from +0.20 to −1.00 V on **a** Co-CNTs and **b** Co-CNTs-MW samples, respectively. **c** The charge density redistribution (charge transfer) of Co-N_2_, Co-N_3_, and Co-N_4_ sites, respectively. Color code: Gray represents carbon atoms; Blue represents nitrogen atoms; pink represents cobalt atoms. The Cyan (yellow) region shows the electron loss (gain) and all the iso-surface values for these three models were 0.003 e bohr^‒3^. **d** The calculated potential free energy diagrams for CO_2_ electroreduction to CO on Co-N_2_, Co-N_3_, and Co-N_4_ sites, respectively. Inset of **d**: Structural evolution of reaction intermediates on representative coordinatively unsaturated Co-N_3_ site in electrochemical CO_2_RR. Notes: solution environment and 6 × 6 unit cell are considered in the above data.

To gain deep insights into the CO_2_RR mechanism over the coordinatively unsaturated Co-N sites, in situ ATR-IR was employed to monitor the evolution of absorbed reaction intermediates from +0.20 to −1.00 V in CO_2_-saturated 0.1 M KHCO_3_ (the in situ test device was shown in Fig. S27). According to the different coordination structures, Co-CNTs-MW (coordinatively unsaturated) and Co-CNTs (coordinatively saturated) were selected as experimental samples. One major peak could be explicitly discerned from the serial IR spectra between 1200 and 1500 cm^−1^. The band at 1379 cm^−1^ is attributed to the C-O stretching mode of *COOH^53–56^. The obvious emergence of potential-dependent *COOH peak for Co-CNTs-MW (−0.70 V) is obviously earlier than that of Co-CNTs (−1.00 V), which indicates that unsaturated Co-N sites feature lower *COOH formation energy than the saturated Co-N sites (Fig. 5a, b)^56,57^. The above in situ ATR-IR results highlight the advantages of coordinatively unsaturated Co-N sites for facilitating the generation of key *COOH intermediates for CO_2_-to-CO conversion.

In order to verify the mechanism of coordinatively unsaturated Co-N sites in enhancing CO_2_-to-CO electroreduction performance further, DFT calculation was carried out (see more details in Supplementary Information) based on different Co-N_x_ sites (x = 2, 3, and 4) on graphited carbon support (Fig. S28). The stability calculations (Fig. S29) indicate that within the reduction potential, the unsaturated Co-N_2_ and Co-N_3_ will not undergo coordination structure transformation. Then, the adsorptions of CO_2_ on Co-N_x_ sites were first examined. The unsaturated Co-N_x_ sites (x = 2 and 3) show lower adsorption energies (0.40 eV for Co-N_2_, 0.35 eV for Co-N_3_) of CO_2_ than the saturated Co-N_4_ site (0.72 eV), implying the better activation process of CO_2_ on unsaturated Co-N_x_ sites (x = 2 and 3). To further understand the better activation function of coordinatively unsaturated Co-N sites, the differential charge diagram was calculated. From Fig. 5c, a few electrons exchange between Co-N_4_ and CO_2_ molecules can be found on the saturated Co-N_4_ site, representing the difficulty of CO_2_ activation. For comparison, with the introduction of N defects, coordinatively unsaturated Co-N_x_ sites (x = 2 and 3) can transfer more electrons from the Co site to activate CO_2_ molecules, corresponding to 0.20|e| for Co-N_2_ and 0.45|e| for Co-N_3_, respectively (Fig. 5c). Further orbital analysis (Fig. S30) shows that the higher HOMO level of Co-N_3_ greatly reduces the gap with the LUMO level of the adsorbed molecule CO_2_ (e.g., −0.90 eV), which facilitates the easier electron transfer. The coordinatively unsaturated Co-N sites could further promote the protonation of CO_2_ into *COOH, which is the rate-determining step on the saturated Co-N_4_ site (Fig. 5d). In addition, Unsaturated nitrogen coordination can also inhibit HER and promote the selectivity of CO (Fig. S31). Generally, the coordinatively unsaturated Co-N sites promote the catalytic activity for CO_2_-to-CO conversion, which is well consistent with in situ ATR-IR results.

### Universality for rapid synthesis of metal-nitrogen sites and gram-scale fabrication

To verify the universality of this microwave-assisted method to prepare efficient CO_2_-to-CO catalysts, Fe-CNTs-MW, Ni-CNTs-MW, and Cu-CNTs-MW were further fabricated via a similar procedure. Both samples exhibit more than 90% FE_CO_ at the optimized potential region in the H-type cell (Figs. S32,  S33a). In order to produce a C_2+_ product, we expanded the loading amount of copper (Cu-cluster). In the flow cell test of 50 mA cm^−2^, 16.6% C_2_H_5_OH and 19.9% C_2_H_4_ were detected (Figs. S33b,  S34). The formation of multi-carbon products may mainly come from Cu nanoparticles on CNTs (Figure S35), which further shows that the microwave method can be applied for the preparation of well-dispersed Cu nanoparticles for multi-carbon products. We also anticipate that further local structure modification over this well-dispersed Cu-cluster catalyst can achieve higher selectivity of C_2+_ products.

In addition, gram-scale fabrication of Co-CNTs-MW (2.5 g) was carried out to prove the effectiveness for large-scale fabrication, and their CO_2_RR selectivity (Fig. S36) was evaluated, only showing a slight decrease in FE_CO_ (~90%) at the optimized working potential of −0.75 V.

## Discussion

In summary, we have developed a simple, fast, and scalable procedure to prepare coordinatively unsaturated M-N-C catalysts for industrial-current CO_2_ electrolysis. The Co-CNTs-MW catalyst delivers high-efficiency CO_2_-to-CO conversion in MEA with FE_CO_ of 95.4% and FCEE of 54.1% at the *j* of 200 mA cm^−2^, which is among the best of CO_2_-to-CO electrolyzers. Moreover, the catalyst can deliver amperage-current CO_2_ electrolysis with applied current up to 10 A, showing a considerable SPC of 40.4%, and drives a continuous CO_2_ reduction process at 2 A for more than 60 h. In situ ATR-IR characterization and DFT calculations demonstrate that CO_2_ molecules can be easily activated and the formation energy of *COOH can be simultaneously lowered over the coordinative unsaturated Co-N sites, which have been identified by AC-STEM and XAFS characterizations. We believe this work broadens an avenue to controllably and massively fabricate atomically dispersed metal-nitrogen active sites for real-world high-rate CO_2_-to-CO electrolysis.

## Methods

### Materials

Potassium hydroxide (KOH), hydrochloric acid (HCl), nitric acid (HNO_3_), sulfuric acid (H_2_SO_4_), and n-butanol were purchased from Sinopharm Chemical Reagent Co., Ltd. Analytical grade cobalt (II) chloride hexahydrate (CoCl_2_·6H_2_O), iron (III) chloride hexahydrate (FeCl_3_·6H_2_O), nickel (II) chloride hexahydrate (NiCl_2_·6H_2_O), copper chloride dihydrate (CuCl_2_ ∙ 2H_2_O), nickel nitrate hexahydrate (Ni(NO_3_)_2_ ∙ 6H_2_O), ferric nitrate monohydrate (Fe(NO_3_)_3_ ∙ 9H_2_O), potassium bicarbonate (KHCO_3_), and isopropanol were obtained from Shanghai Chemical Reagent Co. Ltd. Multi-walled carbon nanotubes (CNTs) was bought from Shanghai Macklin Biochemical Co., Ltd. 1,10-phenanthroline monohydrate was bought from Aladdin Co., Ltd. Nafion (5 wt%) solutions were purchased from Sigma-Aldrich. H_2_IrCl_6_ ∙ 6H_2_O was obtained from Alfa Aesar Co. Ltd. Two kinds of gas-diffusion layers (GDL, H14C9, and Sigracet 28 BC) and anion exchange membranes (Fumasep FAB-PK-130 and Sustainion x37-FA) were purchased from Fuel Cell Store. High-purity argon (Ar) and carbon dioxide (CO_2_, 99.9999%) gases were purchased from the Shanghai Jiajie Special Gas Co., Ltd. All chemicals were used directly without further purification and all water used for synthesis and analysis was purified to >18.2 MΩ·cm by Millipore system.

### Pretreatment of CNTs

For the synthesis of Co-CNTs, the carbon nanotubes (CNTs) were treated in 1.0 M HCl for 24 h to remove the impurities. In order to introduce hydroxyl function groups, the CNTs were hydroxylated with an acid mixture (HNO_3_ (69%) + H_2_SO_4_ (90%) with a volumetric ratio of 1:3) at 60 °C for 2 h. The resultant CNTs were washed with DI water until neutral and was then centrifuged and dried at 60 °C.

### Synthesis of Co-CNTs

A mixture of CoCl_2_ ∙ 6H_2_O (24 mg) and 1,10-phenanthroline monohydrate (60 mg) was dissolved in 10 mL ethanol followed by stirring for ~20 min at room temperature. The pretreated CNTs (136 mg) were added to the above solution. After stirring for 30 min, the solution was centrifuged and dried at 60 °C for 12 h.

### Synthesis of Co-CNTs-800 °C

The dried Co-CNTs were put into a tubular furnace and calcined under a nitrogen atmosphere of 800 °C for 2 h. Co-CNTs-800 °C was obtained by grinding the calcined powder.

### Synthesis of Co-CNTs-MW and Co-CB-MW

The dried Co-CNTs were put into a 10 mL quartz glass bottle and argon gas was introduced for 10 min to remove the air in the bottle. The quartz glass bottle was immediately moved into a household microwave (2.45 GHz, 1.0 kW) and treated for ~2 min. After 5 min of cooling, Co-CNTs-MW was obtained. Similarly, Co-CB-MW were obtained by replacing CNTs with carbon black.

### Synthesis of Fe-CNTs-MW, Ni-CNTs-MW, Cu-CNTs-MW, and Cu-cluster

Fe-CNTs-MW, Ni-CNTs-MW, and Cu-CNTs-MW samples were prepared using a similar procedure to that described above for Co-CNTs-MW, except that the amount of 1,10-phenanthroline monohydrate and metal salt were adjusted as required to achieve to a ratio of 3:1 (1,10-phenanthroline: metal precursor). For the synthesis of Cu-cluster, we changed the centrifugal drying in the synthesis step to static drying, and the other synthesis steps remained unchanged.

### Gram-scale fabrication of Co-CNTs-MW

For the synthesis of Co-CNTs-MW on a large scale, CoCl_2_ ∙ 6H_2_O (300 mg) and 1,10-phenanthroline monohydrate (900 mg) were dissolved in 350 mL of ethanol followed by stirring at room temperature. About 2.55 g pretreated CNTs were added. The remaining operation was similar to that of Co-CNTs-MW preparation.

### Preparation of working electrode

For the H-type cell, 10 mg catalyst, 5 mg carbon black, and 80 μL Nafion solution (5 wt%, Sigma-Aldrich) were mixed in 1 mL isopropanol and sonicated for 30 min. Then 100 μL of the homogeneous ink was drop-casted onto the carbon paper (1 × 1 cm^2^).

For flow cell and MEA configuration, we deposited 10 mg catalyst, 5 mg carbon black mixed with 80 μL Nafion solution (5 wt%) in 2 mL isopropanol, and sonicated for 30 min to form ink solution and then deposited onto GDL (H14C9 in flow cell, Sigracet-28 BC in MEA) using air-brush (mass loading: 1 mg cm^−2^).

### Preparation of NiFe-LDH/NF anode in MEA

The NiFe-LDH/NF was prepared via an electrodeposition method^58^. The electrodeposition was carried out in a standard three-electrode electrochemical cell containing nickel foam (NF) as the working electrode, a parallel-positioned platinum plate as the auxiliary electrode, and Ag/AgCl (3.5 M KCl) as the reference electrode. The electrolyte contained 3 mM Ni(NO_3_)_2_ ∙ 6H_2_O and 3 mM Fe(NO_3_)_3_ ∙ 9H_2_O. The constant potential electrodeposition was then carried out at −1.00 V (vs. Ag/AgCl) for 300 s. After electrodeposition, the NF was carefully withdrawn from the electrolyte, rinsed with water and ethanol, and then sonicated briefly in ethanol and left dry in the air.

### Preparation of IrO_2_/Ti anode in MEA

The IrO_2_/Ti anode was prepared by thermal decomposition method^59^. A titanium felt substrate (100 mm width, 100 mm length, and 1.0 mm thickness) was immersed into the coating solution, which was prepared by dissolving 649 mg of H_2_IrCl_6_ ∙ 6H_2_O into a mixture of 10 mL DI water and 20 mL of concentrated HCl. Firstly, the titanium substrate was immersed in the solution for 5 min, then dried at 120 °C for 30 min, and finally calcined at 500 °C for 60 min. These steps were repeated five times.

### Electrochemical measurements

The electrochemical measurements were conducted in a customized gastight H-type glass cell separated by Nafion 117 membrane. CHI660E electrochemistry workstation was employed to record the electrochemical response. Ag/AgCl electrode (3.5 M KCl) and Pt mesh were used as reference electrodes and counter electrodes, respectively. All potentials in the study were calibrated to the reversible hydrogen electrode (RHE) according to the equation E_RHE_ = E_Ag/AgCl_ + 0.059 × pH + 0.205. All the potentials and voltages in this work were not *iR*-corrected. For the H-type cell test, the electrolyte was 0.5 M KHCO_3_ (30 mL for each compartment) and saturated with high-purity CO_2_ (99.9999%) for at least 30 min before testing (20 sccm, calibrated by mass flow controller). LSV curves were collected with a scan rate of 10 mV s^−1^. EIS measurement was conducted by applying an AC voltage with an amplitude of 5 mV in a frequency range from 10^5^ to 0.001 Hz at a certain overpotential.

For the flow cell test, Co-CNTs-MW was air-brushed onto H14C9 GDL as a CO_2_RR cathode (0.7 × 0.7 cm^2^). Nickel foam (2 × 2 cm^2^) and Ag/AgCl electrode (3.5 M KCl) were used as the counter electrode and reference electrode, respectively. The reference electrode chamber and anode chamber were separated by the Fumasep FAB-PK-130 membrane. On the cathode side, the gas flow rate of CO_2_ is 40 sccm while the anode was circulated with 1.0 M KOH electrolyte at 10 mL min^−1^ flow rate.

For the MEA test in the alkaline solution, Co-CNTs-MW was air-brushed onto Sigracet 28 BC GDL as a CO_2_RR cathode (1 × 1.2 cm^2^) and the NiFe-LDH/NF was used as anode (1 × 1.2 cm^2^). The cathode chamber and anode chamber were separated by the Sustainion x37-FA membrane. On the cathode side, the gas flow rate of CO_2_ is 40 sccm while the anode was circulated with 1.0 M KOH electrolyte at 10 mL min^−1^ flow rate.

For the amperage-current test in MEA sized of 100 cm^2^ in the neutral condition of 0.1 M KHCO_3_, E36233a DC power supply was used for high-current analysis. IrO_2_ (2 mg cm^−2^) loaded on titanium felt was chosen as an anode, and Co-CNTs-MW (1.0 mg cm^−2^) was air-brushed on Sigracet 28 BC GDE as a CO_2_RR cathode. The cathode and anode were separated by a Sustainion x37-FA anion exchange membrane. On the cathode side, under the current of 1, 3, and 5 A, the inlet flow rate is 80 sccm and the outlet flow rate is 72, 60, and 52 sccm respectively. At 8 and 10 A currents, considering the rapid consumption of CO_2_, we expand the CO_2_ flow rate to 150 sccm, and the outlet flow rates are 113 and 102 sccm respectively. The anode was circulated with 0.1 M KHCO_3_ electrolyte at a 20 mL min^−1^ flow rate.

### CO_2_RR products analysis

The gas phase products after bulk electrolysis were quantified by online gas chromatograph (RAMIN, GC2060), equipped with the flame ionization detector (FID for CO and hydrocarbons) and a thermal conductivity detector (TCD for H_2_). The liquid products were quantified using ^1^H nuclear magnetic resonance (NMR) (Varian 700 MHz spectrometer (16.4 T)).

The Faradaic efficiency for CO and H_2_ were calculated based on the equation:

2 $${{{{{{\mathrm{FE}}}}}}}=\frac{2{Vp{{{{{\mathrm{GF}}}}}}}}{{I{{{{{\mathrm{RT}}}}}}}}$$

Where V is the volume concentration of CO or H_2_ in the exhaust gas from the electrochemical cell (GC data). I (mA) is the steady-state total current, G is the CO_2_ flow rate at room temperature and ambient pressure. The constants were shown as follows: *p* = 1.013 × 10^5^ Pa, *T* = 273.15 K, *F* = 96485 C mol^−1^, *R* = 8.3145 J mol^−1^ K^−1^.

### Evaluation of TOF

The TOF for CO was calculated as follows^60^:

3 $${{{{{{\mathrm{TOF}}}}}}}\,\left({h}^{-1}\right)=\frac{{j}_{{{{{{{\mathrm{Co}}}}}}}}/({{{{{{\mathrm{NF}}}}}}})}{{m}_{{{{{{{\mathrm{cat}}}}}}}}\times w/{M}_{{{{{{{\mathrm{Co}}}}}}}}}\times 3600$$

*j*_CO_: partial current (A) for CO product;

N: the number of electrons transferred for product formation, which is 2 for CO;

F: 96485 C mol^−1^;

m_cat_: catalyst mass in the electrode, g;

w: Co loading in the catalyst;

M_Co_: atomic mass of Co, 58.93 g mol^−1^.

### Evaluation of full-cell energy efficiency (FCEE)

FCEE has been calculated via the following formula^44^:

4 $${{{{{{\mathrm{FCEE}}}}}}}=\frac{{{{{{{{\mathrm{FE}}}}}}}}_{{{{{{{\mathrm{CO}}}}}}}}\times {E}^{0}}{{E}_{{{{{{{\mathrm{cell}}}}}}}}}$$

where E^0^ is the equilibrium cell potential for CO production (E^0^ = E^0^_cathode_ - E^0^_anode_ = −0.10 V − 1.23 V = −1.33 V), E_cell_ is the overall cell voltage, and FE_CO_ is the Faradaic efficiency for CO_2_-to-CO conversion.

### Evaluation of time of CO formation and electricity of CO consumed

Time of CO formation (h mol^−1^) has been calculated via the following formula:

5 $${{{{{{\mathrm{Time}}}}}}}\,{{{{{{\mathrm{of}}}}}}}\,{{{{{{\mathrm{CO}}}}}}}\,{{{{{{\mathrm{formation}}}}}}}=\frac{N\times {N}_{A}}{{I}_{{CO}}\times 3600\times 6.24146\times {10}^{18}}$$

N: the number of electrons transferred for product formation, which is 2 for CO;

N_A_: Avogadro constant.

Electricity of CO consumed (kW·h mol^−1^) has been calculated via the following formula:

6 $${{{{{\rm{Electricity}}}}}}\; {{{{{\rm{of}}}}}}\; {{{{{\rm{CO}}}}}}\; {{{{{\rm{consumed}}}}}}=\frac{{E}_{{{{{{{\mathrm{cell}}}}}}}}\times I}{1000}\times \,{{{{{{\mathrm{Time}}}}}}}\,{{{{{{\mathrm{of}}}}}}}\,{{{{{{\mathrm{CO}}}}}}}\,{{{{{{\mathrm{formation}}}}}}}$$

### Evaluation of SPC

7 $${{{{{\rm{Single}}}}}}\,{{{{{\rm{pass}}}}}}\,{{{{{\rm{conve}}}}}}{{{{{\rm{r}}}}}}{{{{{\rm{sion}}}}}}=\frac{{{{{{{{\mathrm{CO}}}}}}}}_{2}\,{{{{{{\mathrm{consumption}}}}}}}\,{{{{{{\mathrm{per}}}}}}}\,{{{{{{\mathrm{minute}}}}}}}}{{{{{{{{\mathrm{CO}}}}}}}}_{2}\,{{{{{{\mathrm{inflow}}}}}}}\,{{{{{{\mathrm{per}}}}}}}\,{{{{{{\mathrm{minute}}}}}}}}$$

### In situ infrared absorption spectroscopy

The in situ attenuated total reflectance infrared spectroscopy (ATR-IR) was measured in a homemade cell (Fig. S24). The cell was connected to a PerkinElmer spectrum 100 spectrometer. The cell contains a complete three-electrode system and a CO_2_ gas inlet and outlet. The catalyst powder deposited on carbon paper acts as the working electrode (WE), in close contact with the silica prism surface. For electrochemical tests, 0.5 M KHCO_3_ saturated with high-purity CO_2_ (99.9999%) was used as the electrolyte solution. A CHI1242C constant potential meter was used for stepwise testing from open circuit voltage (OCV) to −1.00 V vs. RHE with a dwell time of 1 min for each potential.

### Computational details

All the spin-polarized calculations were performed using the Vienna Ab-initio Simulation Package (VASP) package^61–64^. The exchange-correlation function was described by the Perdew–Burke–Ernzerhof (PBE) functional^61^ within the generalized gradient approximation (GGA)^65^. The project-augmented wave (PAW) method^66^ was employed to treat core electrons, and the cutoff energy of the plane-wave basis was set to 450 eV. In addition, the Hubbard *U* correction for *3d*-orbital electrons is used to avoid the insufficient consideration of GGA in the repulsion between spin electrons. The *U* value of 3.42 eV was used for the Co metal center^67^, which provides more reliable calculation results.

We constructed an N-doped graphene substrate (CN), anchoring a single Co atom in the center. For the sake of comparison, the coordinated N atoms were sequentially removed to form N_4_, N_3_, and N_2_ coordinated structures respectively (shown in Fig. S25). A vacuum layer of 15 Å was applied to separate each periodic unit cell (6 × 6). The Brillouin zone was sampled by 2 × 2 × 1 Monkhorst–Pack mesh k-points for all structure optimizations.

### Gibbs free energy (ΔG)

The whole CO_2_RR process involves the transfer of two pairs of H^+^ and e^–^:

8 $${{{{{\mathrm{C}}}}}}{{{{{{\mathrm{O}}}}}}}_{2}(g)+\ast+{H}^{+}({{{{{\mathrm{aq}}}}}})+{e}^{-}\to*{{{{{\mathrm{COOH}}}}}}$$

9 $$\ast {{{{{\mathrm{COOH}}}}}}+{{{{{{\mathrm{H}}}}}}}^{+}({{{{{\mathrm{aq}}}}}})+{e}^{-}\to*{{{{{\mathrm{CO}}}}}}+{{{{{{\mathrm{H}}}}}}}_{2}{{{{{\mathrm{O}}}}}}$$

10 $$\ast {{{{{\mathrm{CO}}}}}}\to {{{{{\mathrm{CO}}}}}}(g)+\ast$$

The adsorption energies of all intermediates are calculated according to the formula: E_a_ = E_ads+slab_ – E_slab_ – E_ads_, where E_ads+slab_, E_slab_, and E_ads_ represent the total energy of the intermediate adsorbed on the surface, the energy of the clean surface and the energy of the adsorbate in the gas/liquid phase, respectively. Furthermore, the Gibbs free energy change (ΔG) during the whole CO_2_ reduction process can be defined as ΔG = ΔE + ΔZPE – TΔS + Δsol (pH = 0 and T = 298 K), where ΔE, ΔZPE, ΔS are the DFT calculated total energy difference, zero-point energy difference and entropy difference, respectively. To consider the effect of the solution environment, we employed the implicit CANDLE solvation model^68^ by JDFTx software upon all structures, using the Garrity–Bennett–Rabe–Vanderbilt (GBRV) ultrasoft pseudopotentials (USPP)^69^. We corrected the solvation energy (Δsol) resulting from JDFTx into Gibbs free energy to eliminate the effect of the solution environment.

### Characterizations

The crystal structure of the samples was examined by X-ray diffraction (XRD, D/max2550V). Raman analysis was carried out by using a Leica DMLM microscope (Renishaw) with the 514 nm laser. SEM characterization was performed using a scanning electron microscope (Hitachi S4800). Aberration-corrected STEM characterization was performed on a Thermo Fisher Themis Z microscope equipped with two aberration correctors under 300 kV. High-angle annular dark-field (HAADF)-STEM images were recorded using a convergence semi-angle of 11 mrad, and inner- and outer collection angles of 59 and 200 mrad, respectively. Energy-dispersive X-ray spectroscopy (EDS) was carried out using 4 in-column Super-X detectors. More detailed chemical compositions were collected on X-ray photoelectron spectroscopy (XPS, Thermo Escalab 250) with Al Kα X-ray beam (1486.6 eV), and all binding energies were calibrated using the C 1 *s* peak at 284.8 eV as the reference. Inductively coupled plasma optical emission spectroscopy (ICP-OES) was carried out on a NexION 2000-(A-10) to determine the Co concentration. The surface area of the samples was obtained by the method of Brunauer–Emmett–Teller (BET).

### XAFS measurements and analyses

XAFS data including XANES and EXAFS at Co K-edge were collected at 1W1B beamline in BSRF (Beijing Synchrotron Radiation Facility, China), operated at ~200 mA and ~2.5 GeV. The Co K-edge XAFS measurements were performed in fluorescence mode. The scan range was kept in an energy range of 7509–8509 eV for Co K-edge.

The Co K-edge data were processed by using the ATHENA module implemented in the IFEFFIT software packages. First, subtracting the post-edge background from the overall absorption and normalizing it with respect to the edge jump step to obtain EXAFS spectra is necessary. Then, χ(k) data in the k-space were Fourier transformed to real (R) space using Hanning windows. The fitting of Co K-edge XAFS were obtained using the module ARTEMIS of programs of IFEFFIT^33^. The wavelet transform (WT) was obtained from k^3^-weighted χ(k) signals of Co K-edge based on Morlet wavelets^32^.

## Supplementary information

Supplementary Information

## Data Availability

The data supporting the conclusions of this study are available within the paper and its Supplementary Information. Additional data were available from the corresponding author upon reasonable request. Source data are provided with this paper.

## Source data

Source Data
