# Supplementary material for: Scalable synthesis of coordinatively unsaturated metal-nitrogen sites for large-scale CO2 electrolysis
Source: Nat Commun. 2023 Mar 23;14:1599. doi: 10.1038/s41467-023-36688-6 (PMC10113237; doi:10.1038/s41467-023-36688-6)
Supplement: Supplementary file 1 — Supplementary Information [file 41467_2023_36688_MOESM1_ESM.pdf]

# Supplementary Information

## Scalable synthesis of coordinatively unsaturated metal-nitrogen sites for large-scale $CO_2$ electrolysis

Ji Wei Sun[1], Xuefeng Wu[1], Peng Fei Liu[1]*, Jiacheng Chen[2], Yuanwei Liu[1], Zhen Xin Lou[1], Jia Yue Zhao[1], Hai Yang Yuan[1], Aiping Chen[1], Xue Lu Wang[3], Minghui Zhu[2], Sheng Dai[4]*, and Hua Gui Yang[1]*

[1]Key Laboratory for Ultrafine Materials of Ministry of Education, Shanghai Engineering Research Center of Hierarchical Nanomaterials, School of Materials Science and Engineering, East China University of Science and Technology, 130 Meilong Road, Shanghai 200237, China.

[2]State Key Laboratory of Chemical Engineering, School of Chemical Engineering, East China University of Science and Technology, 130 Meilong Road, Shanghai 200237, China.

[3]Physics Department and Shanghai Key Laboratory of Magnetic Resonance, School of Physics and Electronic Science, East China Normal University, 3663 North Zhongshan Road, Shanghai 200062, China.

[4]Key Laboratory for Advanced Materials and Feringa Nobel Prize Scientist Joint Research Center, Institute of Fine Chemicals, School of Chemistry and Molecular Engineering, East China University of Science and Technology, 130 Meilong Road, Shanghai 200237, China.

*Correspondence and requests for materials should be addressed to Hua Gui Yang (hgyang@ecust.edu.cn), Sheng Dai (shengdai@ecust.edu.cn) and Peng Fei Liu (pfliu@ecust.edu.cn)

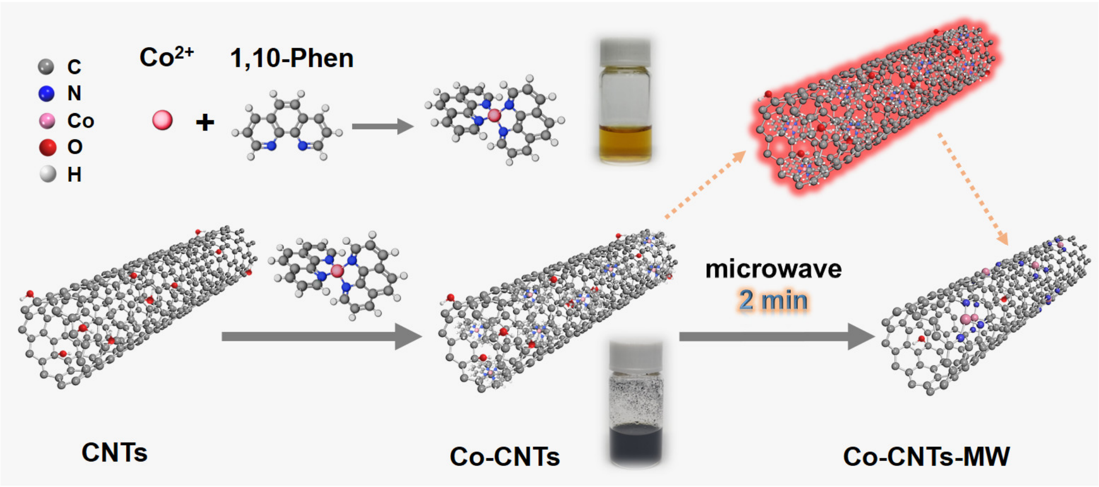


**Supplementary Figure S1.** The schematic illustration of rapid synthesis of typical Co-CNTs-MW prepared by microwave method. 1,10-phen molecules were used to coordinate Co ions for further adsorption on CNTs. The resultant Co-CNTs then underwent rapid microwave treatment within 2 min to obtain Co-CNTs-MW.

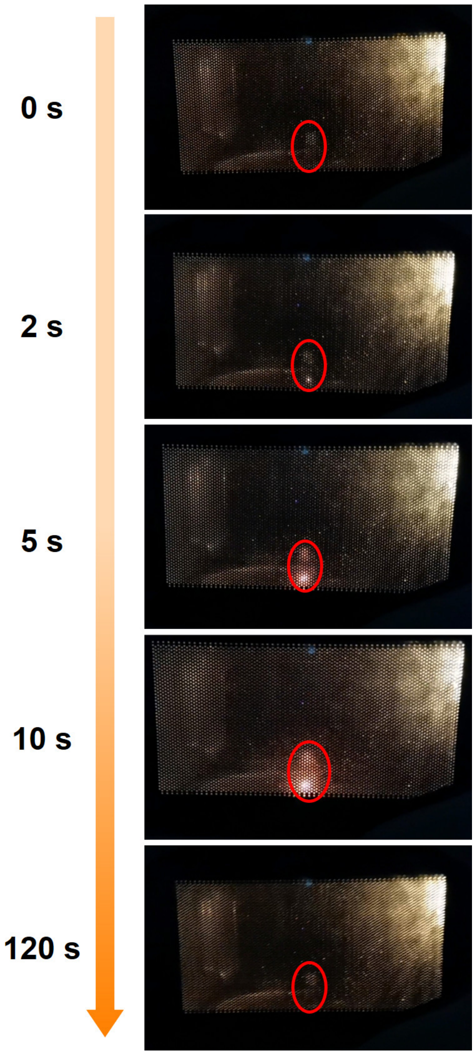


**Supplementary Figure S2.** The photograph taken during the microwave synthesis of Co-CNTs-MW, showing the shining flash during the rapid thermal treatment.

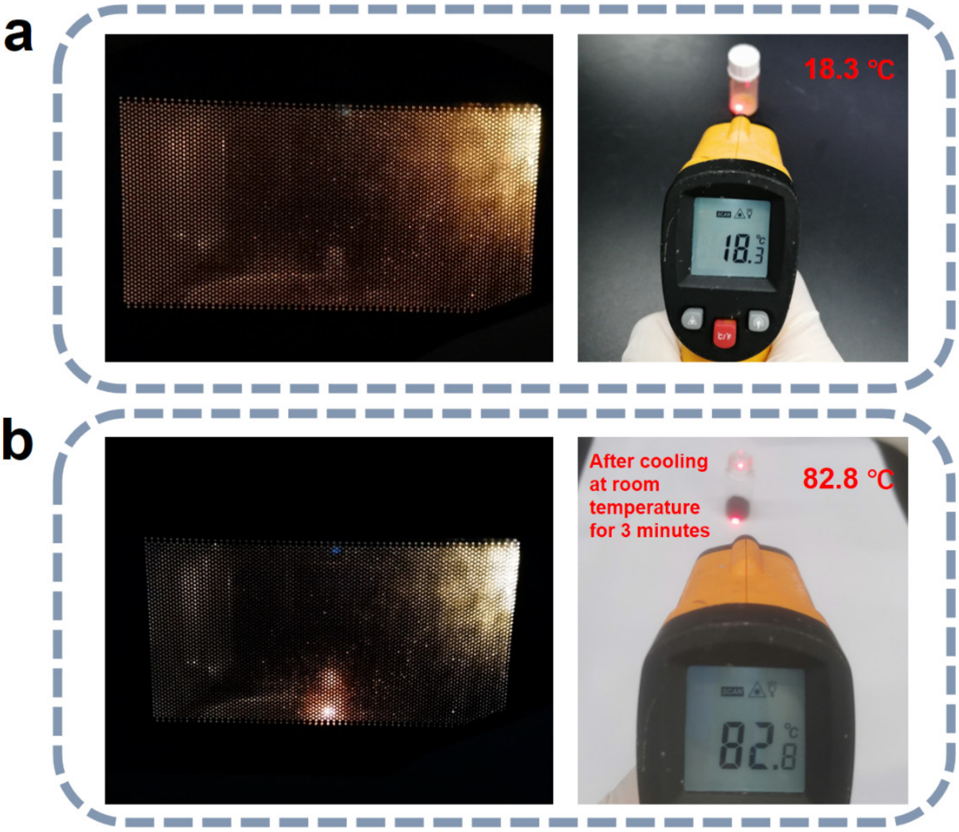


**Supplementary Figure S3.** Temperature comparison chart of (a) cobalt complexes and (b) CNTs after microwave heating. Cobalt complexes were only 18.3 °C after microwave, while CNTs still had 82.8 °C after cooling for three minutes at room temperature.

Notes: For further verification, we prepared pure cobalt complexes. Then cobalt complexes and CNTs were heated by microwave, respectively. No obvious heating phenomenon was observed in the microwave process of cobalt complexes, but obvious heating luminescence was observed in the microwave process of CNTs. The cobalt complex heated by microwave was measured immediately, and it was found that its temperature was only room temperature. The temperature of microwave heated CNTs was still as high as 82.8 °C after cooling at room temperature for three minutes

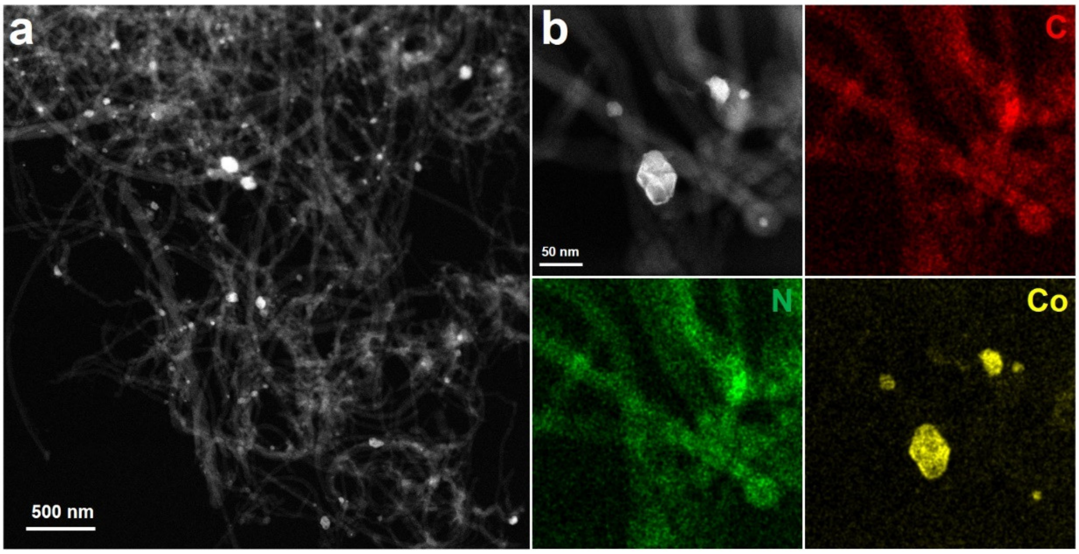


**Supplementary Figure S4.** (a) Typical HAADF-STEM image of Co-CNTs-800 °C and (b) corresponding EDS maps of C, N, and Co elements, showing obvious agglomerated Co particles on the CNTs surface.

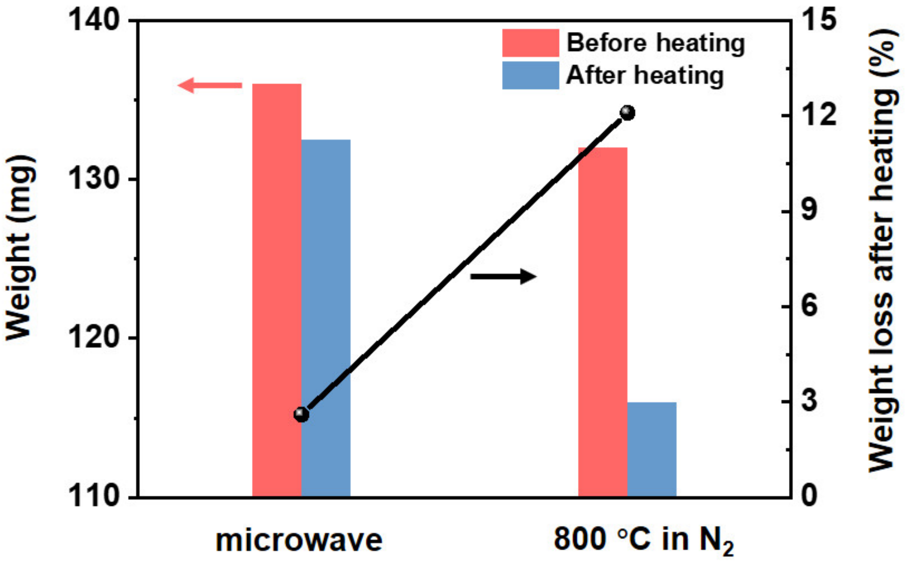


**Supplementary Figure S5.** Comparison of mass loss of catalysts prepared from the same precursor by microwave method and 800 °C calcination in tube furnace. After calcination in a tube furnace at 800 °C for 2 hours under $N_2$, the weight of the catalyst has a 12.1% loss, while the microwave method has only a 2.6% weight loss.

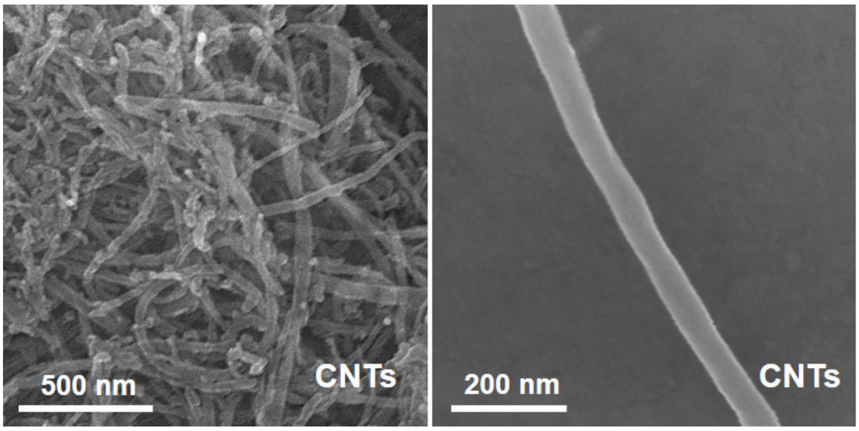


**Supplementary Figure S6.** Typical SEM images of pristine CNTs, which indicates that the microwave treatment would not destroy the morphology of CNTs, combined with the SEM image of Co-CNT-MW.

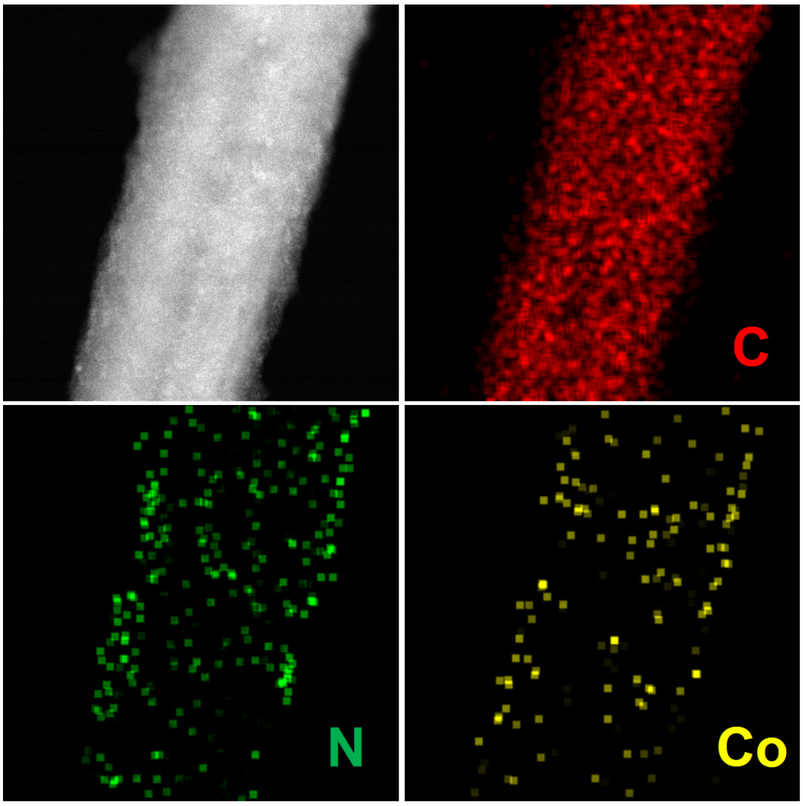


**Supplementary Figure S7.** Typical HAADF-STEM image of Co-CNTs-MW and corresponding EDS elemental maps of C, N, and Co.

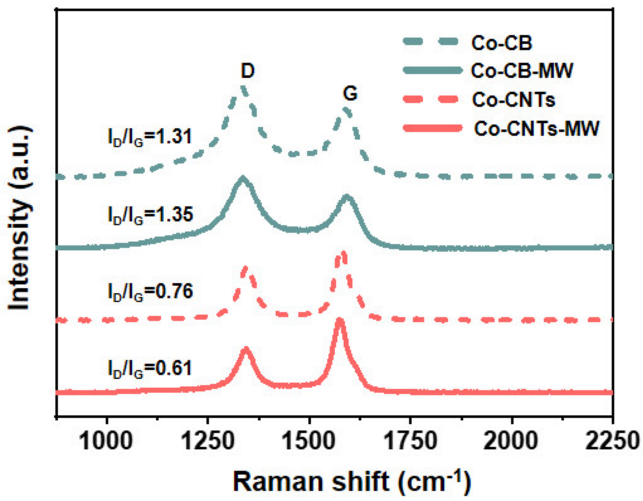


**Supplementary Figure S8.** Raman spectra of Co-CB, Co-CB-MW, Co-CNTs and Co-CNTs-MW, respectively. The carbon black shows more defective carbon structures compared with carbon nanotubes. The microwave irradiation would restore the ordered graphite carbon in Co-CNT-MW.

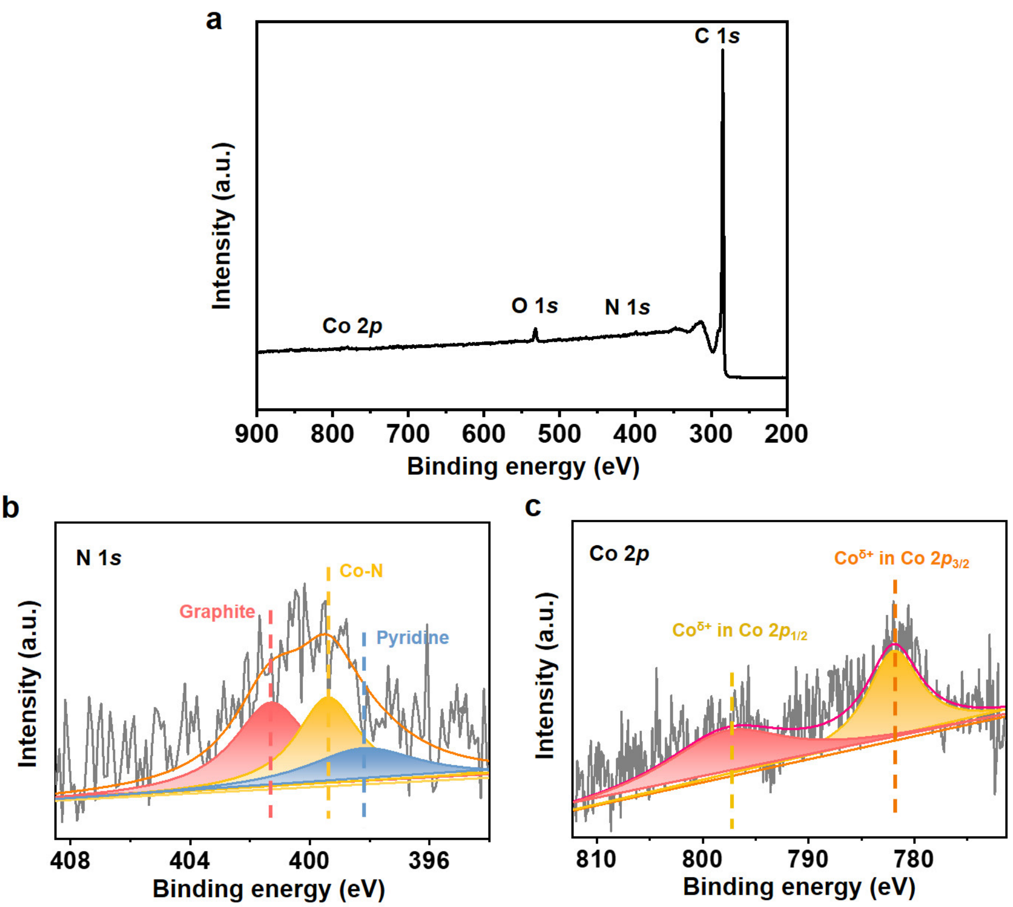


**Supplementary Figure S9.** (a) XPS survey spectrum of Co-CNTs-MW. Only C, O, N, and Co elements were detected. (b) N 1*s* and (c) Co 2*p* XPS spectra of Co-CNTs-MW.

Notes: The N 1*s* spectrum of Co-CNTs-MW was deconvoluted into pyridine N (398.2 eV), Co-N (399.4 eV) and graphitic N (401.4 eV) peaks, respectively. The Co 2*p* spectrum shows the peaks located at 781.1 and 797.4 eV are assigned to oxidized Co species in the Co $2p_{3/2}$ and Co $2p_{1/2}$ regions, respectively.

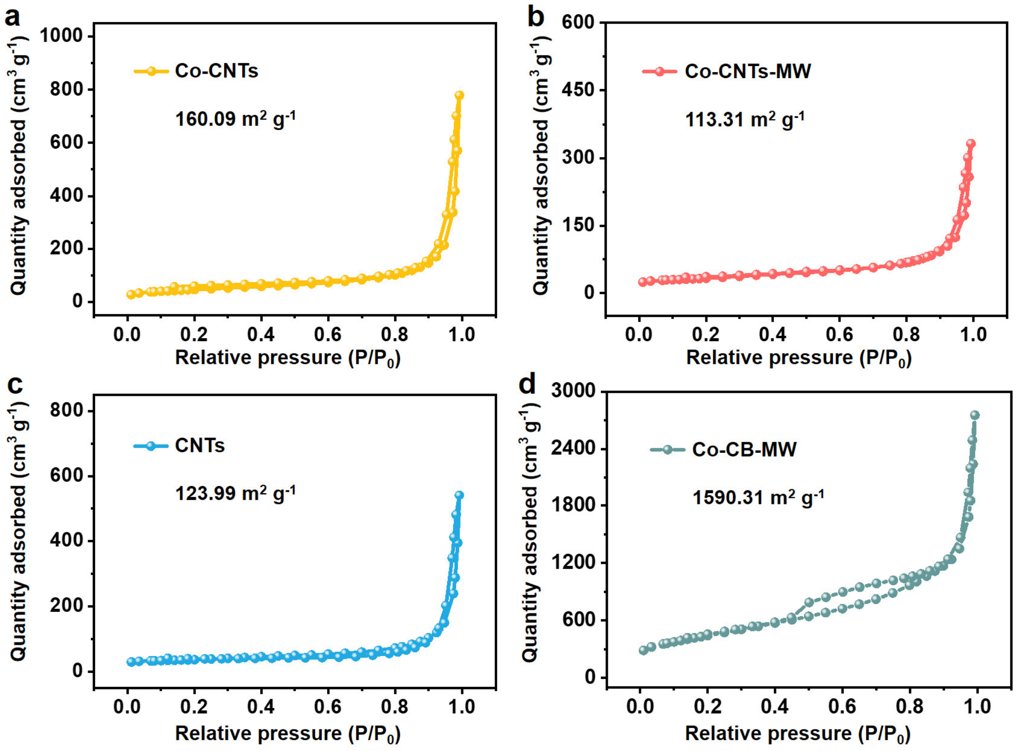


**Supplementary Figure S10.** $N_2$ adsorption-desorption isotherms of (a) Co-CNTs, (b) Co-CNTs-MW, (c) CNTs, and (d) Co-CB-MW for the measurements of BET surface areas.

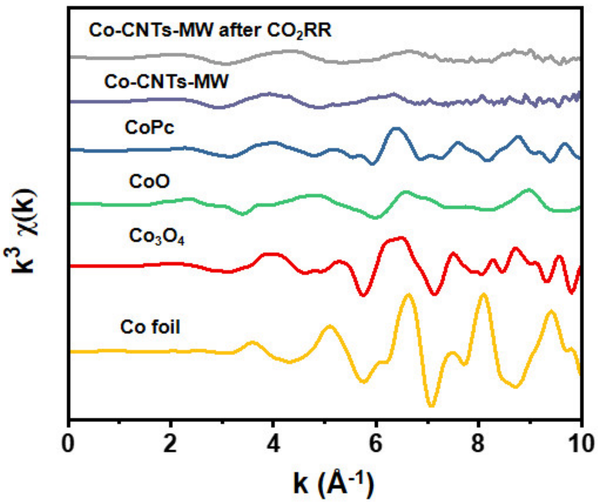


**Supplementary Figure S11.** k-space Co K-edge EXAFS spectra of samples.

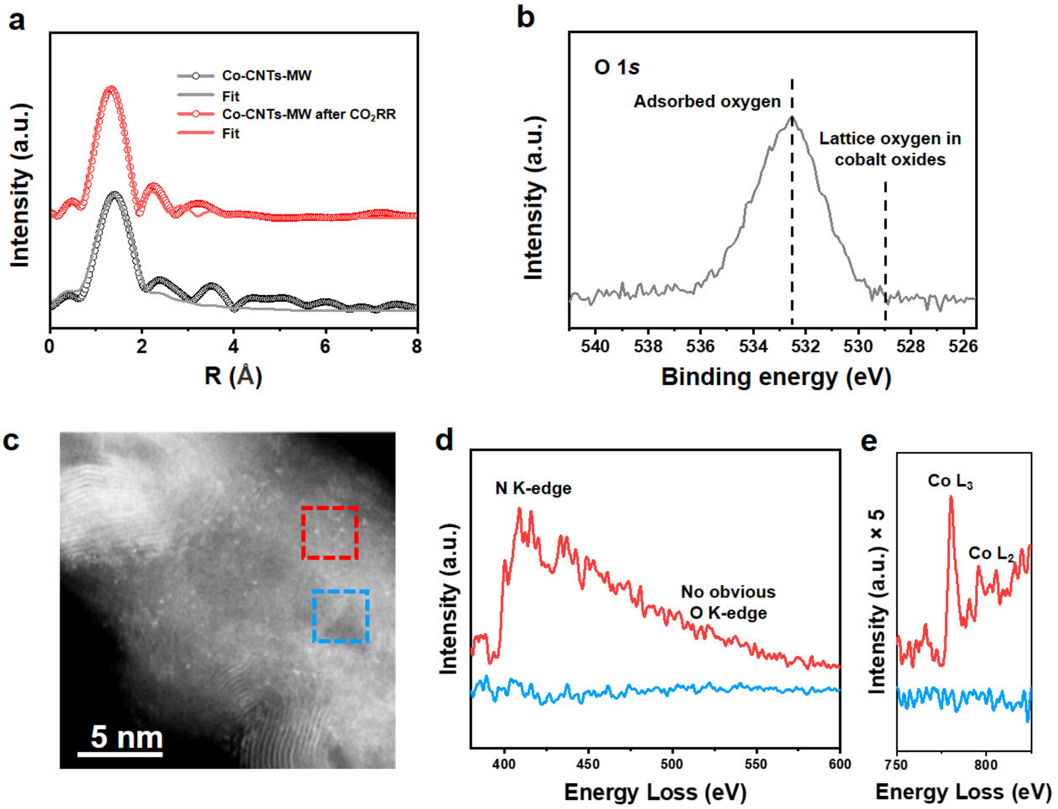


**Supplementary Figure S12.** (a) FT-EXAFS fitting curves of Co-CNTs-MW and Co-CNTs-MW after $CO_2$RR in R space. (b) XPS spectra in the O 1*s* region of Co-CNTs-MW. (c) Atomic-scale HAADF-STEM image of Co-CNTs-MW. (d, e) Electron energy loss spectra acquired from the square regions in (c). The spectrum collected from the red region (containing several Co single atoms) shows an obvious signal of N K-edge and Co L-edges but no O K-edge. The spectrum collected from the blue region (containing no Co atoms) displays no corresponding peaks of N, Co and O.

Notes: XPS spectra in the O 1*s* region of Co-CNTs-MW show that the oxygen in the catalyst exists in the form of adsorbed oxygen (532.6 eV), no lattice oxygen in cobalt oxides was observed near 529 eV.[1] Atomic-scale HAADF-STEM image of Co-CNTs-MW is shown in Figure S12c, in which the Co single atoms can be identified. The EELS spectra were collected from the red and blue regions in Figure S12c, where the red region contains several Co single atoms and the blue region has no Co atom, respectively. The EELS acquisition conditions were same in the two regions, and the

intensity is normalized by the zero loss peak. As shown in Figure S12d, an obvious N K-edge peak can be recognized at 400.2 eV, while no O K-edge signal can be found (around 532.0 eV). Due to the limited Co content, the intensity of the Co L-edges is enlarged 5 times for a better presentation. As shown in Figure S12e, Co $L_3$-edge (780.5 eV) and Co $L_2$-edge (795.2 eV), corresponding to the Co atoms in the red region, are identified. This evidence demonstrates the existence of N around the Co single site and negligible O around Co. The reason why the adsorbed oxygen is not detected is that the unstable adsorbed oxygen is easy to be eliminate by the electron beam illumination under STEM-EELS mode. This also shows that the oxygen on the metal single atom is unstable adsorbed oxygen. In contrast, as for the blue region, no Co single atoms are found, and no Co L-edges peak is recognized. Meanwhile, neither N K-edge nor O K-edge are identified in the EELS spectrum, again demonstrating the point that N around the Co single site.

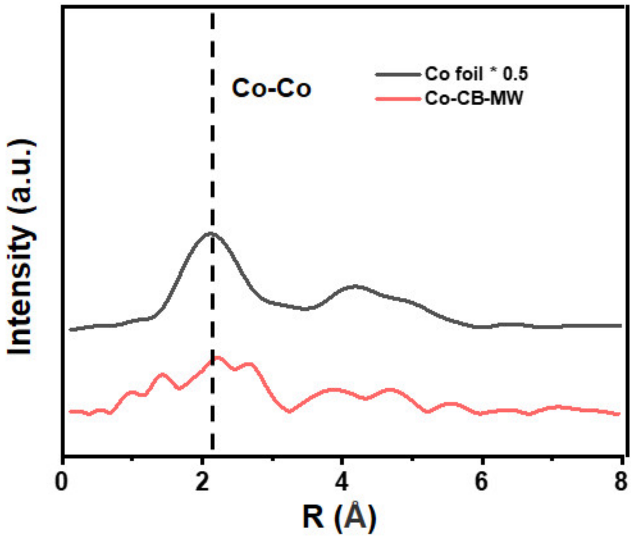


**Supplementary Figure S13**. Co K-edge Fourier transformed EXAFS spectra in the R space of Co foil and Co-CB-MW.

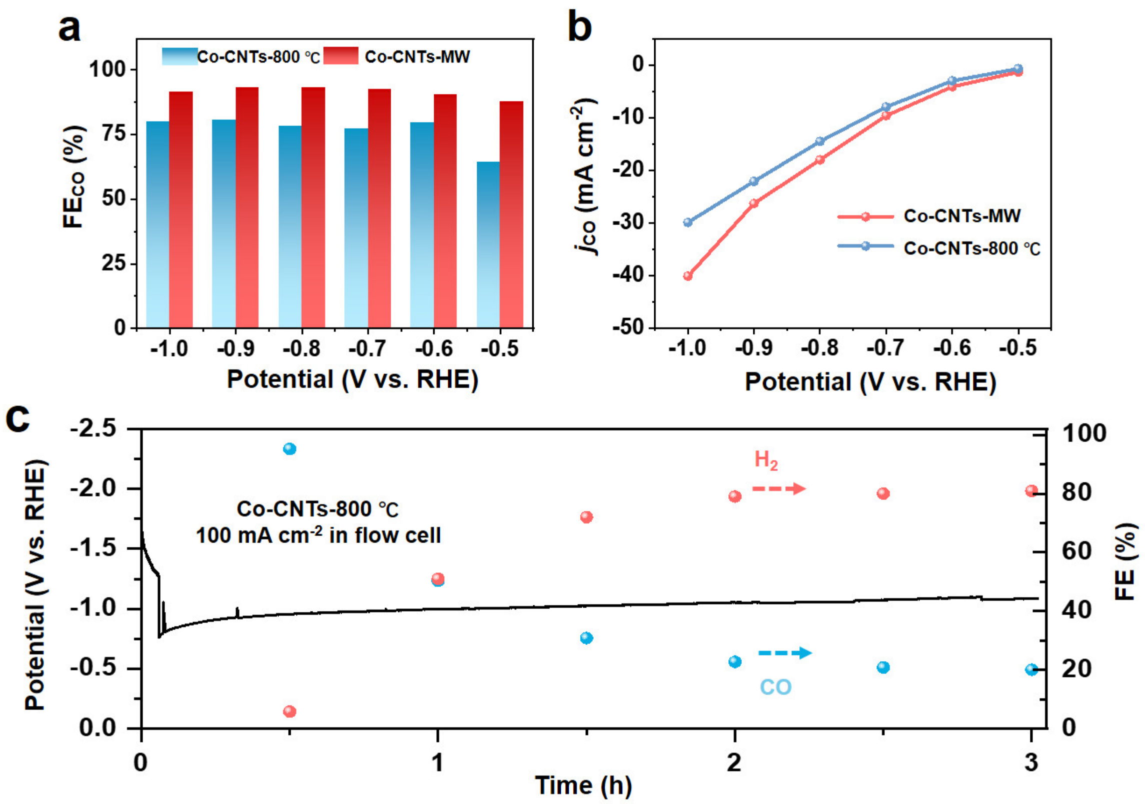


**Supplementary Figure S14.** (a) CO Faradaic efficiencies and (b) CO partial current densities at various applied potentials on Co-CNTs-MW and Co-CNTs-800 °C in H-cell. Co-CNTs-MW has a higher CO selectivity than Co-CNTs-800 °C. (c) Long-term electrolysis under a current density of 100 mA $cm^{-2}$ in flow cell on Co-CNTs-800 °C. Co-CNTs-800 °C exhibited only 20% CO selectivity after 3 hours of testing.

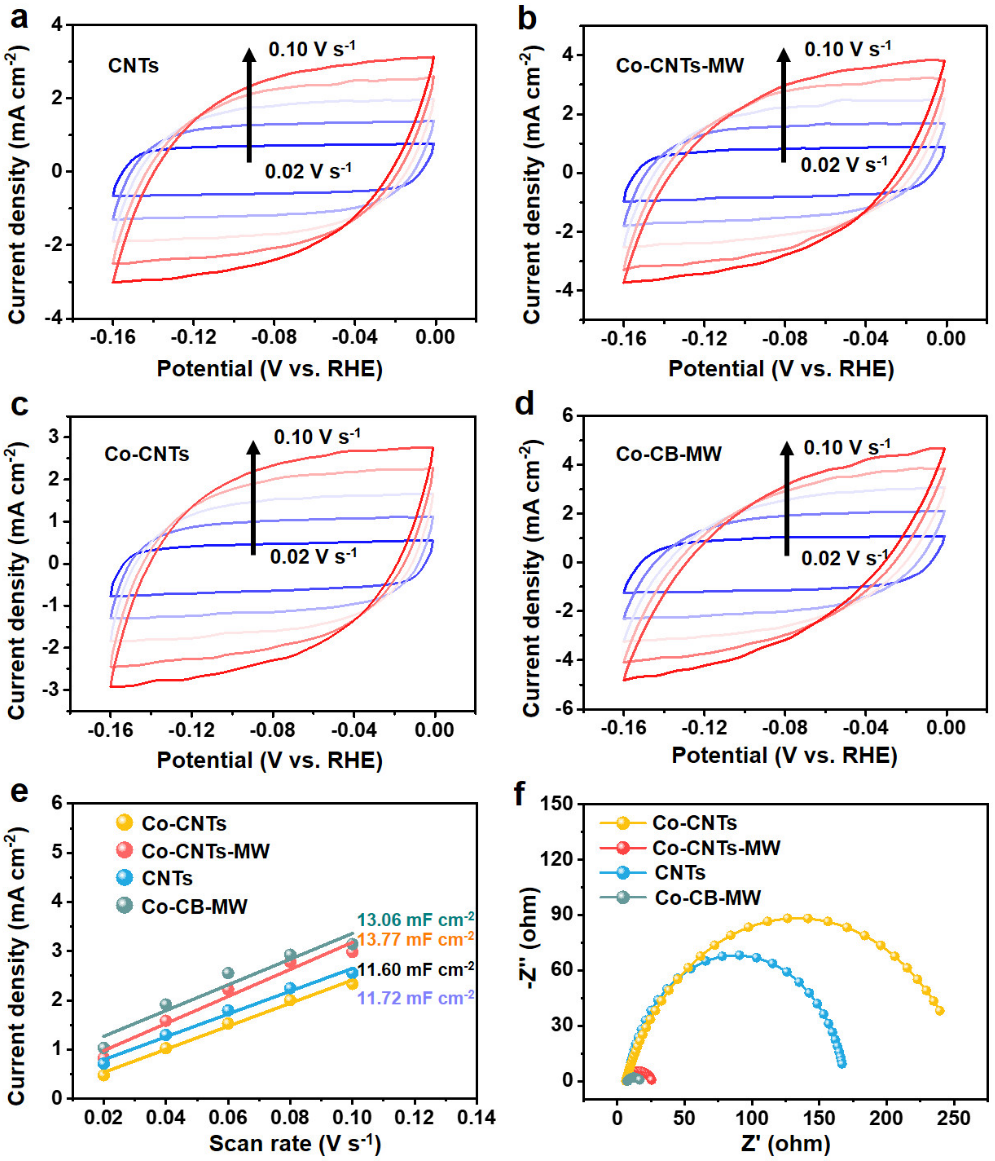


**Supplementary Figure S15.** CV curves with varied scan rates (0.02 to 0.10 V $s^{-1}$) for (a) CNTs, (b) Co-CNTs-MW, (c) Co-CNTs and (d) Co-CB-MW. (e) The capacitive current density plotted as a function of scan rate for the above catalysts, showing no obvious changes in ECSA. (f) Nyquist plots of the above catalysts, exhibiting faster electron transport ability of Co-CNTs-MW and Co-CB-MW after microwave treatment during $CO_2$RR process.

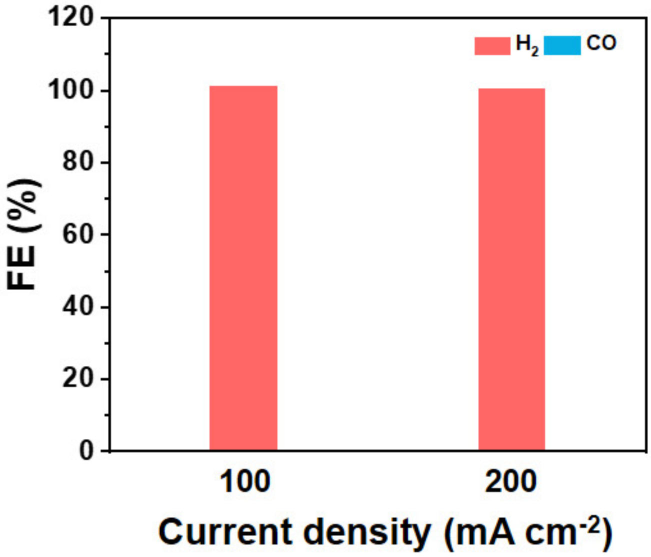


**Supplementary Figure S16.** Selectivity test of Co-CNTs-MW catalyst in the flow cell under Ar, which preliminarily shows that the carbon in the product CO comes from $CO_2$.

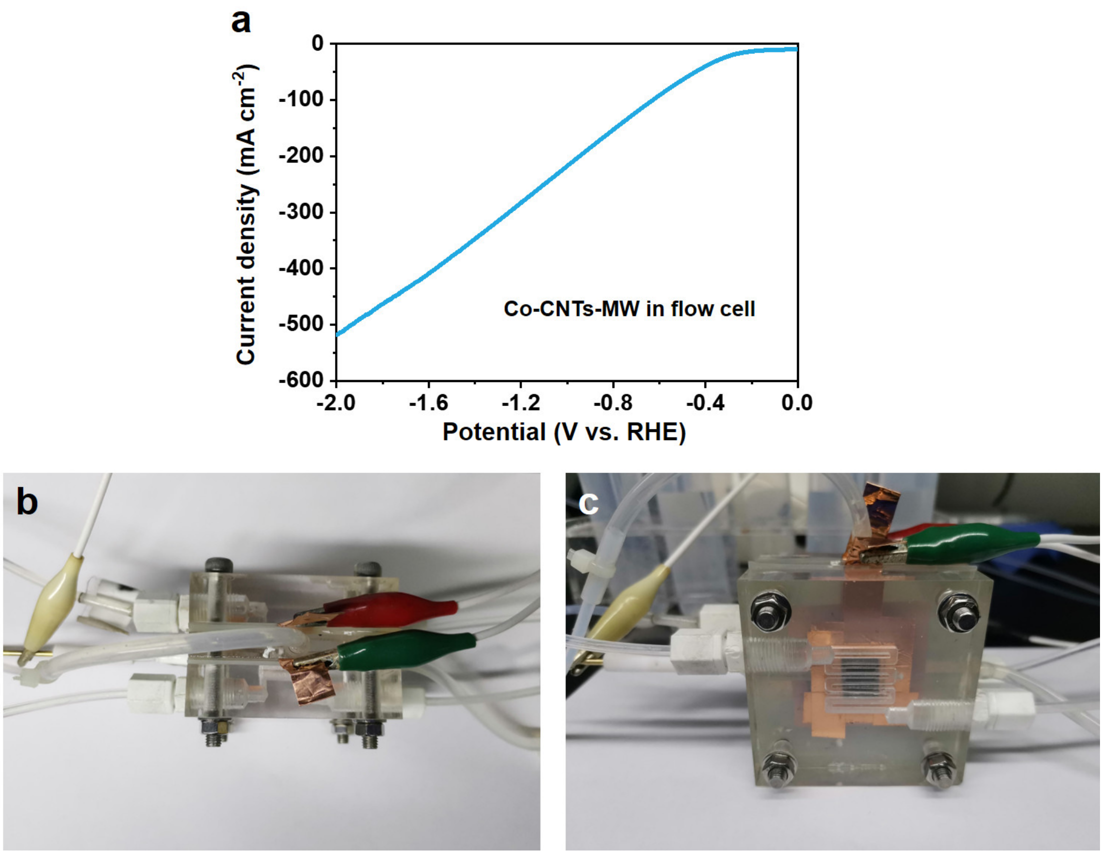


**Supplementary Figure S17.** (a) LSV curve of Co-CNTs-MW acquired in a home-made flow cell (1.0 M KOH). Digital photographs of (b) top-viewed and (c) front-viewed flow cell.

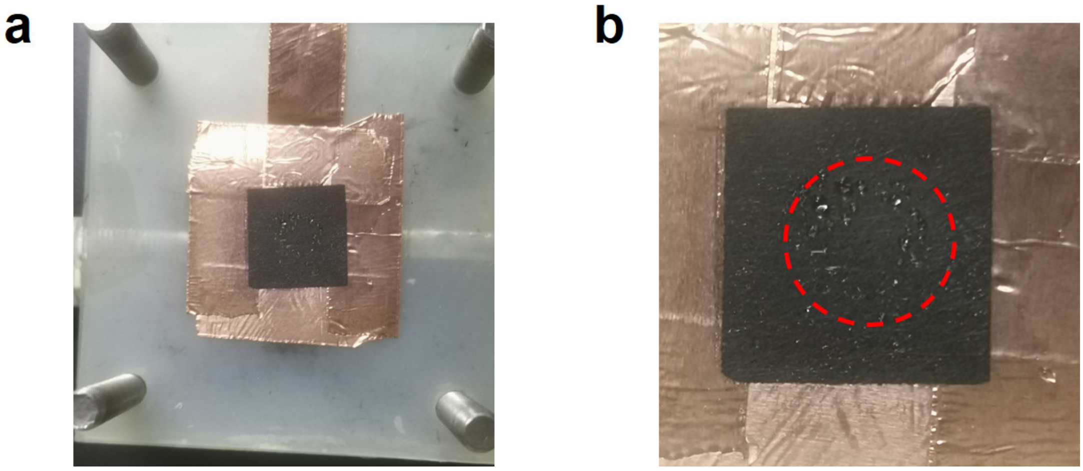

**Supplementary Figure S18.** Digital photos of GDE after 20 hours of testing, which shows that GDE was broken down and obvious water droplets appeared on the back.

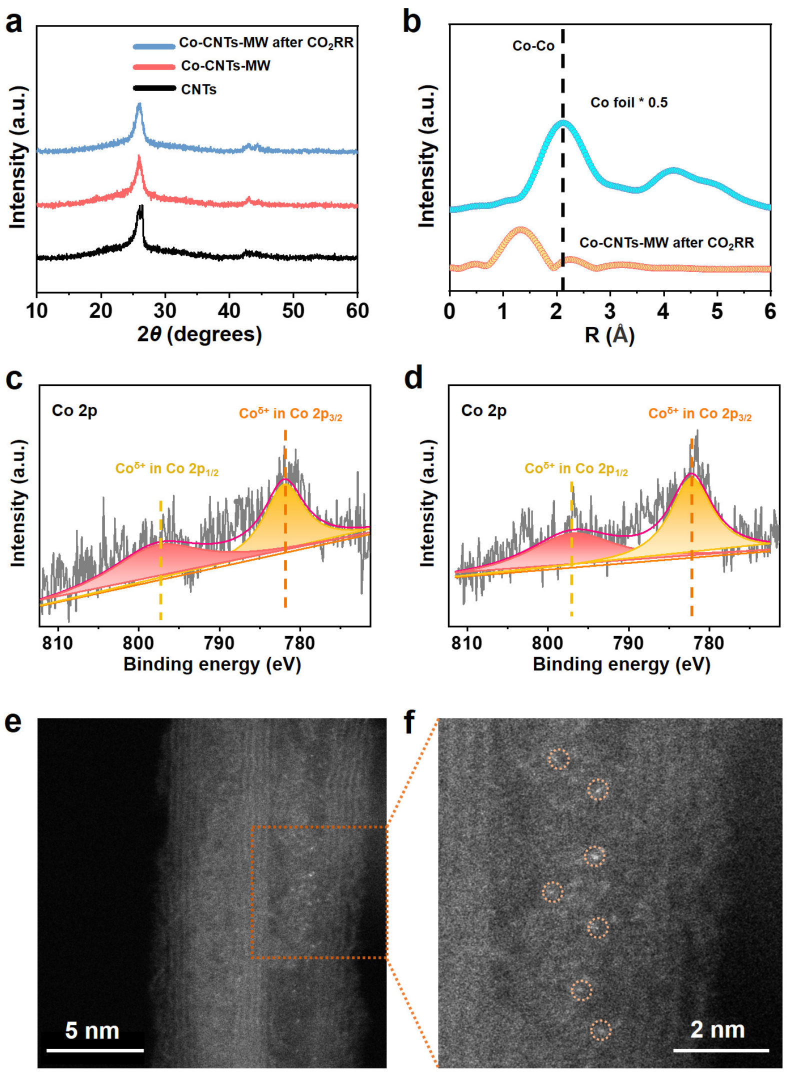


**Supplementary Figure S19**. (a) XRD patterns of CNTs, Co-CNTs-MW and Co-CNTs-MW after $CO_2RR$, respectively, showing no obvious diffraction peaks of crystalline metal or metal oxide species after $CO_2RR$. (b) Co K-edge Fourier transformed EXAFS spectra in the R space of Co foil and Co-CNTs-MW after $CO_2RR$. This further indicates that the catalyst did not produce metal agglomeration after $CO_2RR$. XPS spectra in the Co 2p region of (c) Co-CNTs-MW and (d) Co-CNTs-MW after $CO_2RR$. This indicates that the valence state of Co-CNTs-MW did not change significantly after the reaction. (e, f) Aberration-corrected HAADF-STEM images of Co-CNTs-MW. The atomically dispersed Co sites were marked by orange circles.

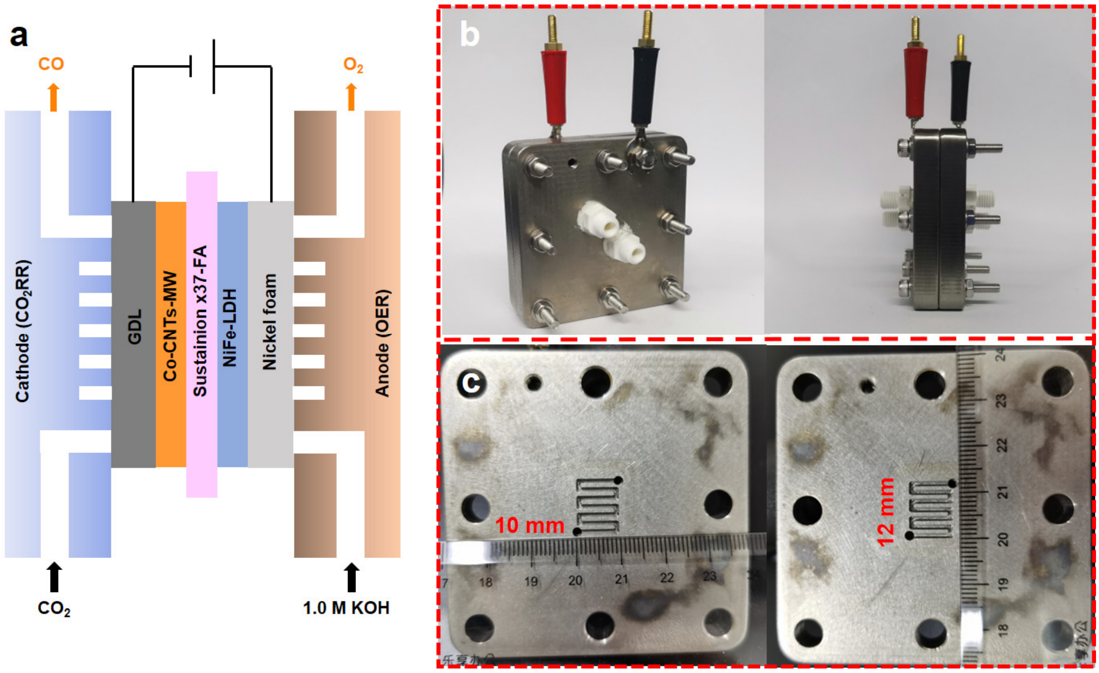


**Supplementary Figure S20.** (a) Schematic diagram of individual part of integrated MEA. (b) Overall digital photographs of MEA. (c) Digital photographs of flow channel size of MEA, showing that the length and width of the flow channel are 12 mm and 10 mm, respectively.

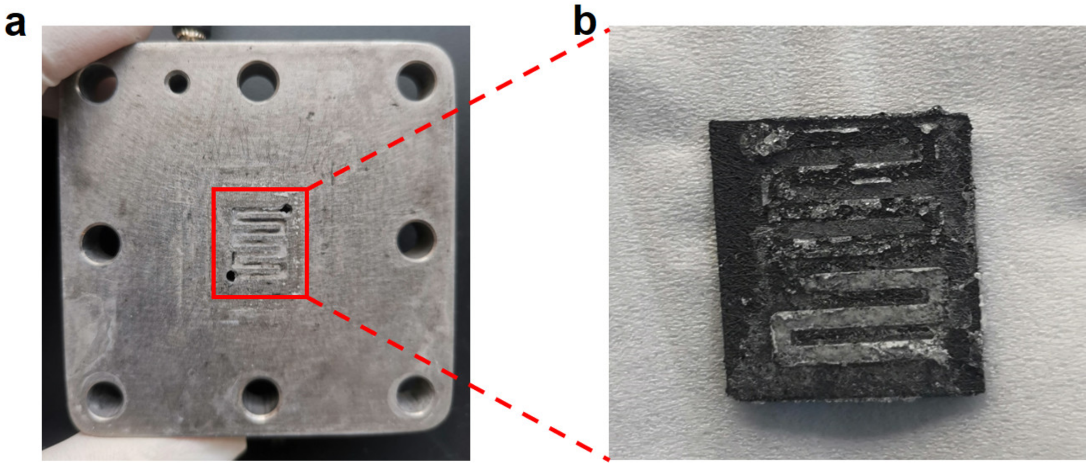


**Supplementary Figure S21.** Long-term $CO_2$ gas diffusion electrolysis under a current density of 100 mA $cm^{-2}$ in MEA (1.0 M KOH), showing that the $FE_{CO}$ is decayed after continuous 10 h electrolysis which might be attributed to the salt accumulation in the alkaline electrolyte. The obvious slat crystallization was observed in (a) the channel of MEA and (b) the back of GDE.

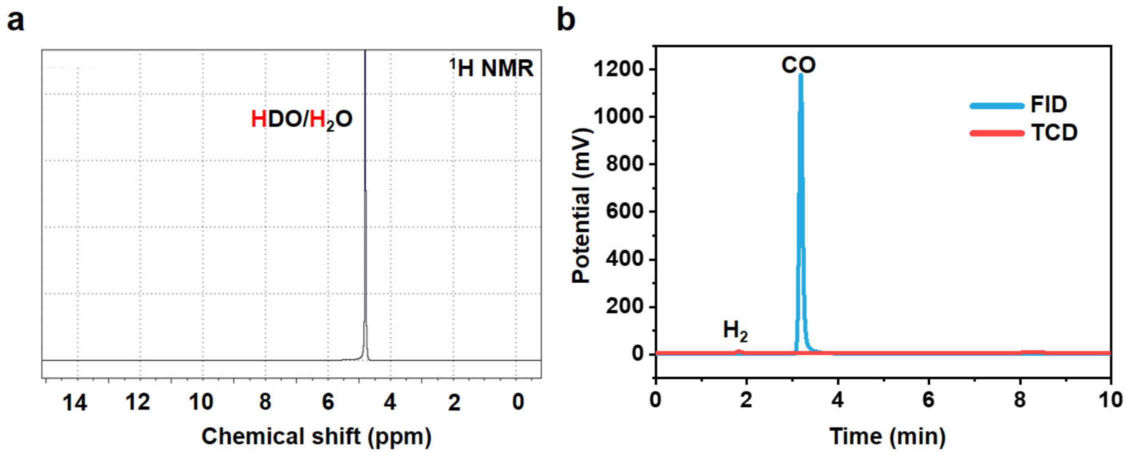


**Supplementary Figure S22.** (a) $^1$H NMR spectrum of the Co-CNTs-MW after 10 h electrolysis at 100 mA cm$^{-2}$ in MEA. No detectable liquid products were produced during the $CO_2$-to-CO conversion process on Co-CNTs-MW catalysts. (b) Typical raw data collected by chromatogram, where FID is the CO detector and TCD is the $H_2$ detector.

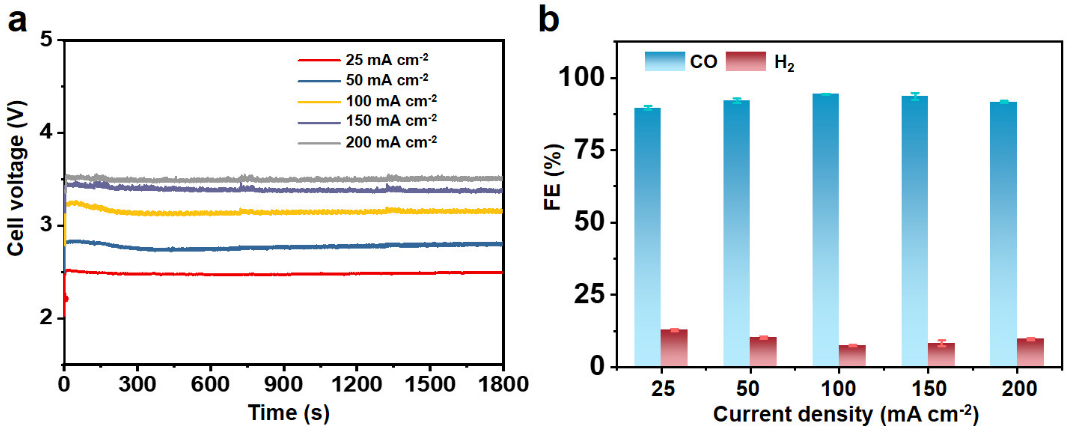


**Supplementary Figure S23.** (a) Cell voltages at different current densities in 1800 s electrolysis in 0.1 M $KHCO_3$ of the MEA sized of 1.2 $cm^2$. (b) CO and $H_2$ Faradaic efficiencies on Co-CNTs-MW catalyst in the range of current densities from 25 to 200 mA $cm^{-2}$ in 0.1 M $KHCO_3$ of the MEA sized of 1.2 $cm^2$.

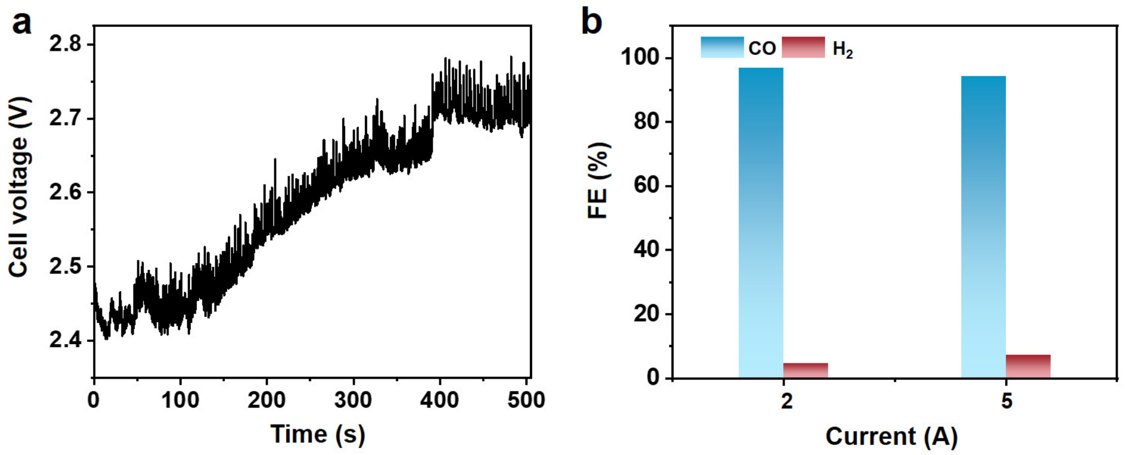


**Supplementary Figure S24.** (a) Cell voltage change in MEA under 2 A current with 1.0 M KOH as electrolyte. The cell voltage has increased by 0.3 V in just 500 seconds. (b) CO and $H_2$ Faradaic efficiencies in MEA under currents of 2 and 5 A with 1.0 M KOH as electrolyte.

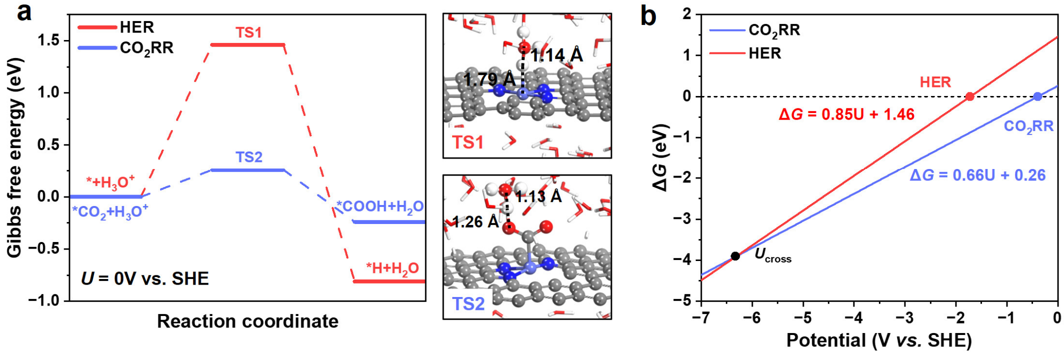


**Supplementary Figure S25** (a) Energy profiles with the activation barriers considered hydrogenation reaction on the unsaturated N-coordinated Co-$N_3$ with corresponding optimized structures of the transition states (TS). (b) Linear relationship (ΔG - U) between ΔG(*$CO_2$→*COOH), ΔG(*H), and potential. The Ucross corresponds to ΔG(*$CO_2$→*COOH) = ΔG(*H), i.e., the turning point of selective hydrogenation.

Notes: As shown in Figure S28a, the energy barrier for HER (1.46 eV) is higher than that for $CO_2$RR (0.26 eV), which visually exhibited the efficient selectivity of catalyst toward $CO_2$RR with an applied potential of 0 V *vs.* SHE. Then we further considered the influence of applied potential on elementary reaction (especially hydrogenation reaction), and calculate the number of electrons transferred from the initial state (IS) structure to the transition state (TS) structure through the constant capacitance model. The results showed that for the same hydrogen proton source, the electrons transfer number of HER (Δq = 0.85 |e|) was greater than that of $CO_2$RR (Δq = 0.66 |e|). This difference in the number of electrons transferred represents the different sensitivity of each reaction to the applied potential. As shown in Figure S28b, we plotted the relationship between Gibbs free energy ($\Delta G$) and potential ($U$) according to the formula of $\Delta G = \Delta q \times U + \Delta G^0$ (where $\Delta G^0$ is the energy barriers calculated on potential 0 V, in Figure S28a). More electrons transferred (i.e., higher slope) caused $\Delta G$(HER) to decrease more rapidly than the hydrogenation reaction in $CO_2$RR as the potential increased, and made the catalyst selectivity change toward HER when the potential exceeds over -6.3 V *vs.* SHE. That is to say, as the potential increases, HER becomes more and more competitive with the yield of $CO_2$RR decreases comparatively, which is consistent with the experimental phenomenon.

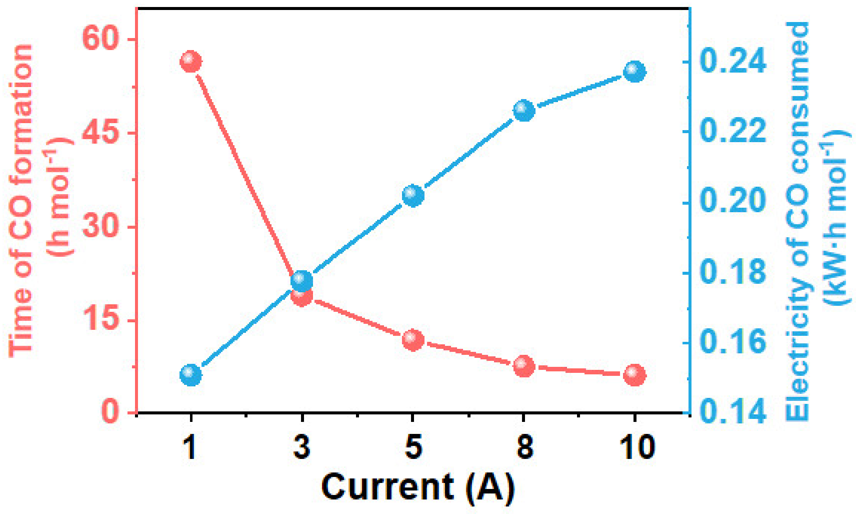


**Supplementary Figure S26.** Time of CO formation and electricity of CO consumed on the Co-CNTs-MW catalyst in MEA sized of 100 $cm^2$.

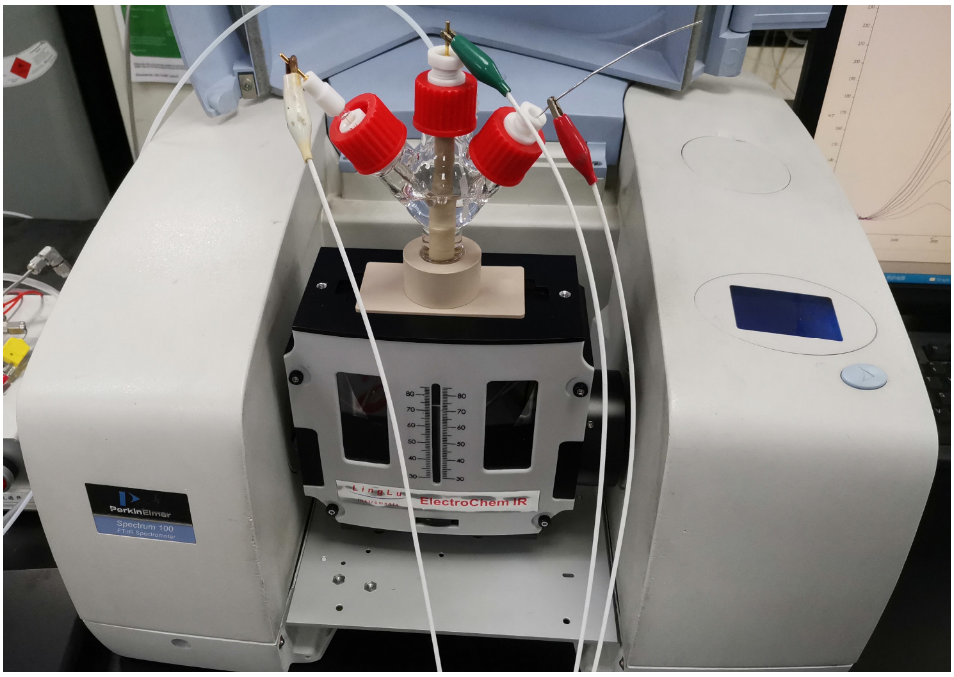


**Supplementary Figure S27.** Digital photograph of *in situ* ATR-IR device.

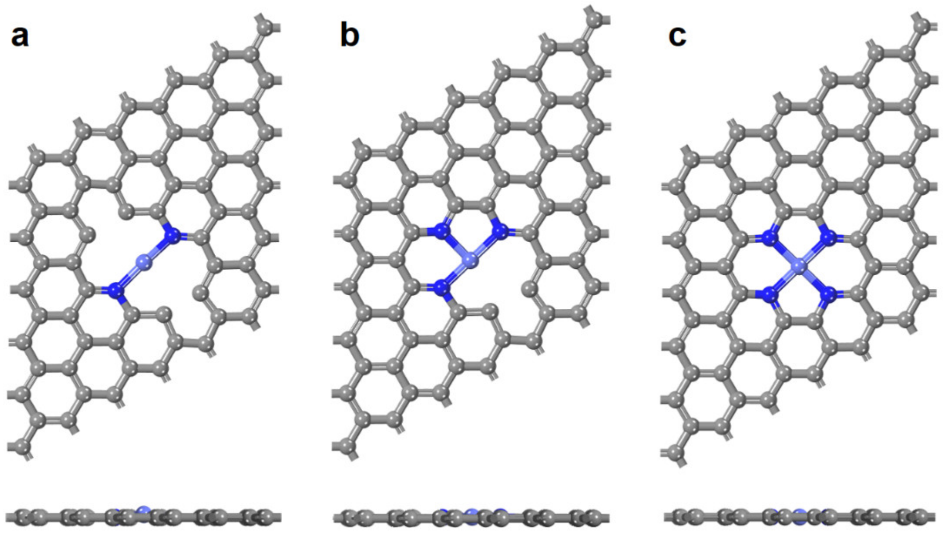


**Supplementary Figure S28.** The 6x6 unit cell structural illustration of atomically dispersed Co-N sites with (a) Co-$N_2$, (b) Co-$N_3$ or (c) Co-$N_4$ configurations, respectively.

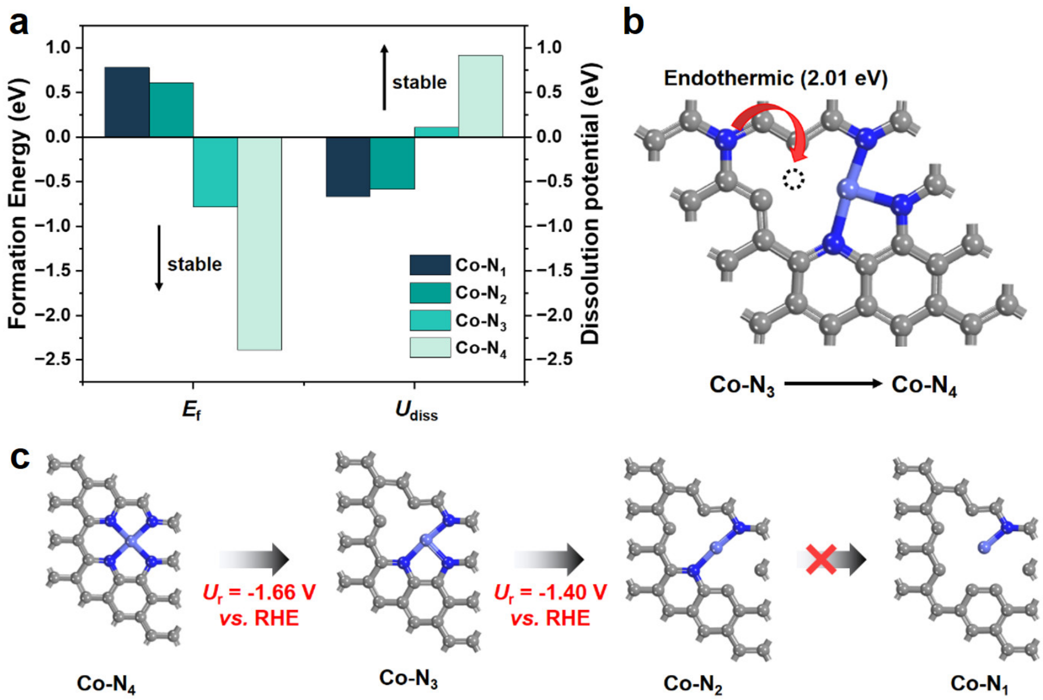


**Supplementary Figure S29**. (a) Computed formation energy and dissolution potential of Co-$N_x$ (x = 1, 2, 3, 4). (b) The conversion of Co-$N_3$ to Co-$N_4$ is endothermic with 2.01 eV. (c) The conversion of different Co-$N_x$ (x =1, 2, 3, 4) with the required applied reduction potential ($U_r$).

Notes: The formation energy ($E_f$) and dissolution potential ($U_{diss}$) of Co-$N_x$ (x = 1, 2, 3, 4) were examined, which can be used to assess the thermodynamic and electrochemical stabilities, respectively. The relevant formulas are as follows:

$$E_f = E_b - E_c = (E_{Co\text{-}CNx} - E_{CNx} - E_{Co}) - E_c \quad (1)$$

$$U_{diss} = U_{diss}^{o} - E_f/(ne) \quad (2)$$

where $E_b$ and $E_c$ are the binding energy of metal center (Co) with substrate and the cohesive energy of metal, respectively; $E_{Co\text{-}CNx}$, $E_{CNx}$, and $E_{Co}$ are the energies of the substrate $CN_x$ with a metal atom anchored, pure substrate $CN_x$ and the Co atom, respectively; $n$ is the number of electrons involved in the dissolution process; $U_{diss}^{o}$ is the standard dissolution potential of metal from experiments. According to our definition, the more negative $E_f$ and more positive $U_{diss}$ mean the more superior thermodynamic and electrochemical stabilities, respectively.

As can be seen from Figure S31a, the thermodynamic and electrochemical stabilities of Co-$N_x$ gradually deteriorate with the continuous reduction of the N coordination number. Although the stability of Co-$N_2$ may not be ideal, the unsaturated

$Co\text{-}N_3$ configuration is still thermodynamically ($E_f$ = -0.78 eV) and electrochemically ($U_{diss}$ = 0.11 eV) stable.

Notably, in the process of DFT structure optimization, the coordination structure of $Co\text{-}N_x$ (x = 2, 3, 4) remained the same. Considering the gradient stability of $Co\text{-}N_x$, we calculated the conversion between different $Co\text{-}N_x$ to illustrate the structural transformation issue in the optimization process. In the experiment, only 1, 10-phenanthroline was used as a nitrogen source, which was decomposed due to the high temperature during the microwave process, leaving the nitrogen element anchored to the carbon nanotubes. Therefore, in the calculation of $Co\text{-}N_3$ as an example, we can see that the local structure of $Co\text{-}N_3$ needs to grab the residual N anchored on graphene to saturate the coordination to become $Co\text{-}N_4$. Through thermodynamic calculations, we found that this is an endothermic reaction process (2.01 eV), so $Co\text{-}N_3$ is difficult to come back to $Co\text{-}N_4$ during optimization, as shown in Figure S31b.

In addition, we also examined the reduction of N coordination, as shown in Figure S31c. According to the following reaction (eq-3) and formula (eq-4), we calculated the applied reduction potential ($U_r$) required for transformation between different structures:

$$Co\text{-}N_x + 3(H^+/e^-) \rightarrow Co\text{-}N_{x-1} + NH_3 \quad (3)$$

$$U_r = -\Delta G^0/3e \quad (4)$$

where $\Delta G^0$ is the Gibbs free energy of eq-3 at zero potential.

It is obvious that such a structural transition from high coordination to low coordination was also relatively difficult and requires a higher potential compared with onset potential (*e.g.*, -1.66 V vs. -0.89 V and -1.40 V vs. 0.60 V). Noting that $Co\text{-}N_1$ is difficult to maintain during optimization, so it is almost impossible for $Co\text{-}N_2$ to continue to lose its N coordination to form $Co\text{-}N_1$ with the Co-N topology undestroyed. That is to say, as long as the theoretical reduction potential ($U_r$) is maintained within -1.40 V, the unsaturated coordination structures of $Co\text{-}N_2$ and $Co\text{-}N_3$ will not undergo coordination structure transformation.

In conclusion, the unsaturated coordination structure $Co\text{-}N_3$ has a more efficient activity than $Co\text{-}N_4$ on the premise of maintaining its own stability.

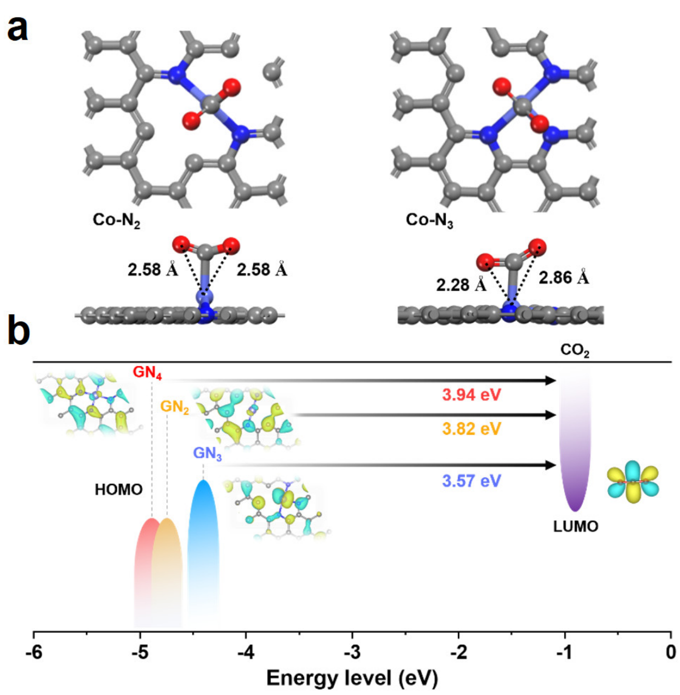


**Supplementary Figure S30.** (a) The optimized adsorption structures of $CO_2$ on Co-$N_2$ (left) and Co-$N_3$ (right) in the vertical view (upper) and front view (down). (b) Calculated HOMOs of Co-$N_x$ (x = 2, 3, and 4) and LUMO of $CO_2$, as well as their relative energy levels.

Notes: In Co-$N_2$, the unsaturated vacancy of N is symmetrically distributed, resulting in the $CO_2$ plane being more perpendicular to the N coordination plane after optimization. It is this symmetrical spatial configuration that gives rise to two Co-O bonds (2.58 Å) of equal length and almost no interaction, meaning that the active site only exchanges electrons with C in $CO_2$ molecule. However, in Co-$N_3$, the single N vacancy causes the $CO_2$ to skew toward the vacancy in the optimization process, which makes the distance between Co and one of the O atoms shorten (2.28 Å) with a certain degree of orbital hybridization, resulting in more electrons can be transferred from Co to $CO_2$ molecule.

The orbital analysis of these species was also performed. The HOMO level of Co-$N_3$ is mainly located at around -4.47 eV, which is more positively shifted compared with that of Co-$N_2$ and Co-$N_4$ (-4.72 eV and -4.84 eV). According to the frontier molecular orbital theory, the higher HOMO level of Co-$N_3$ greatly reduces the gap between it and the LUMO level of the adsorbed molecule $CO_2$ (*e.g.*, -0.90 eV), which facilitates the easier electron transfer from the Co-$N_3$ (0.45 |e|) to $CO_2$ compared with Co-$N_2$ (0.20 |e|) and Co-$N_4$.

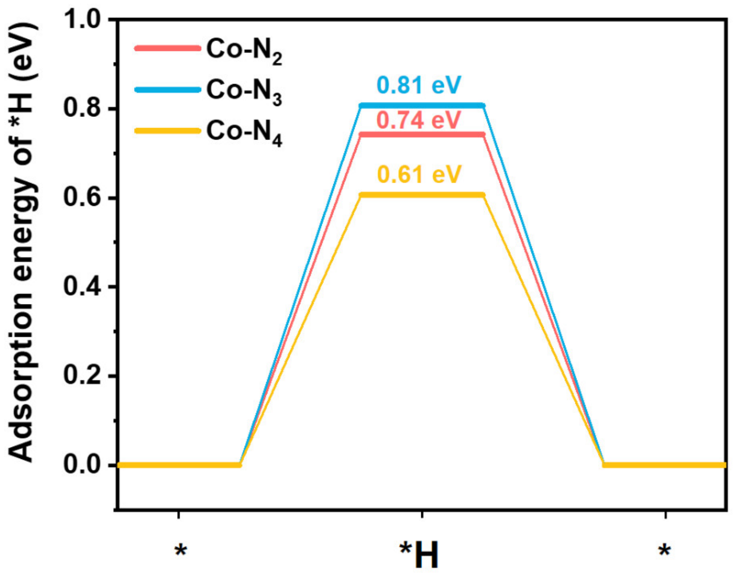


**Supplementary Figure S31.** The calculated adsorption free energy of *H on Co-$N_2$ (0.74 eV vs. 0.64 eV for $CO_2$RR), Co-$N_3$ (0.81 eV vs. 0.73 eV for $CO_2$RR) and Co-$N_4$ (0.61 eV vs. 0.89 eV for $CO_2$RR). Co-$N_4$ is more favorable for HER than Co-$N_2$ and Co-$N_3$.

Notes: Solution environment and 6x6 unit cell are considered in the above data.

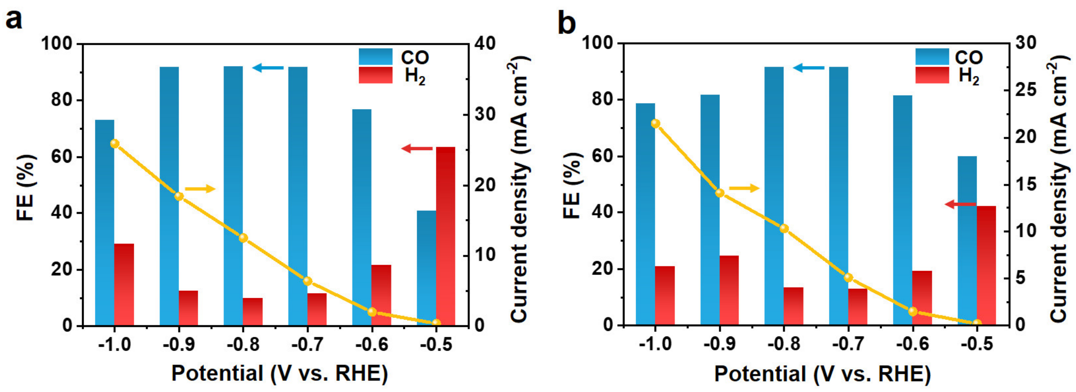


**Supplementary Figure S32.** Potential-dependent $FE_{CO}$ and current density at various applied potentials from -0.50 to -1.00 V on (a) Fe-CNTs-MW and (b) Ni-CNTs-MW, respectively, which were tested in the H-type cell in $CO_2$ saturated 0.5 M $KHCO_3$. Both samples show optimized $CO_2$-to-CO conversion activity, with $FE_{CO}$ over 90% at -0.70 and -0.80 V.

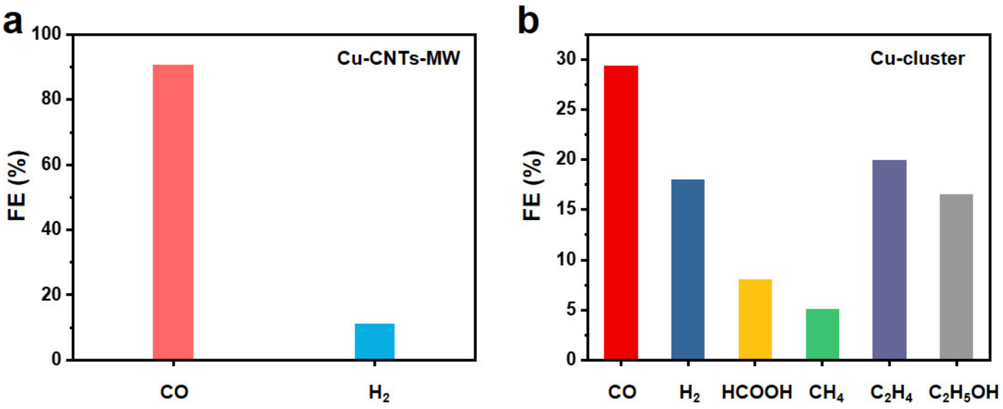


**Supplementary Figure S33.** (a) Faradaic efficiencies at -0.75 V vs. RHE on Cu-CNTs-MW in H-cell. (b) Faradaic efficiencies on Cu-cluster catalyst in the current densities of 50 mA $cm^{-2}$ in the flow cell. With the increase of copper loading, $C_2$ products appeared for the Cu-cluster catalyst.

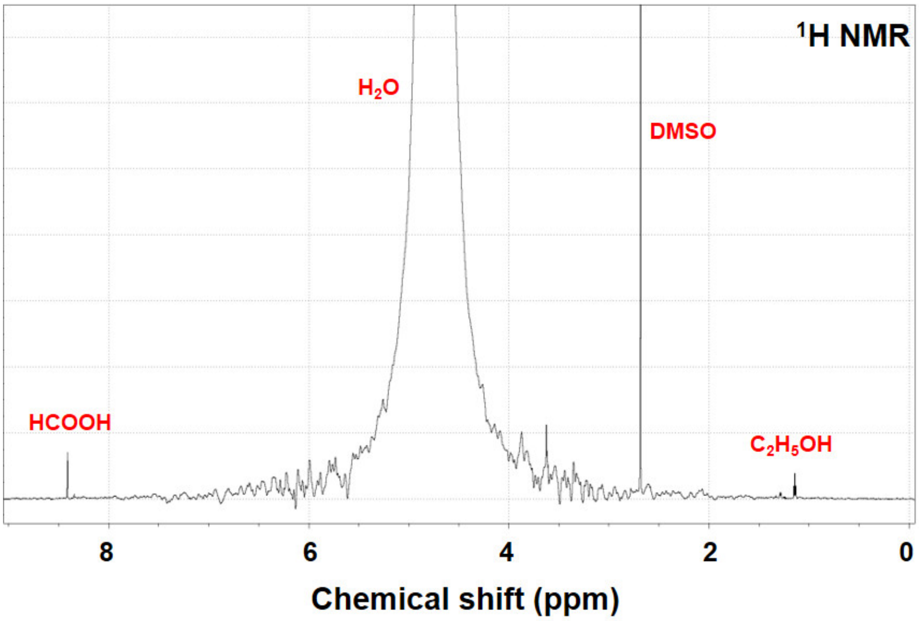


**Supplementary Figure S34.** $^1$H NMR spectrum of the Cu-cluster after 2 h electrolysis at 50 mA cm$^{-2}$ in MEA. HCOOH and $C_2H_5OH$ were detected in the liquid phase products.

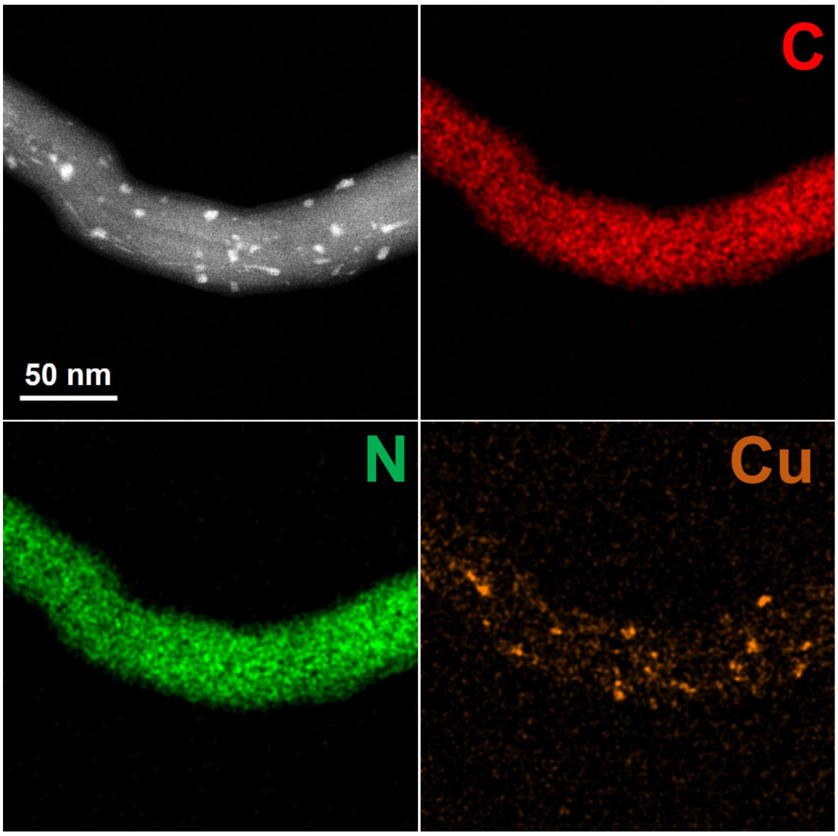


**Supplementary Figure S35.** The HAADF-STEM images and corresponding EDS maps of C, N, and Cu elements, showing obvious agglomerated Cu nanoparticles on the surface of CNTs.

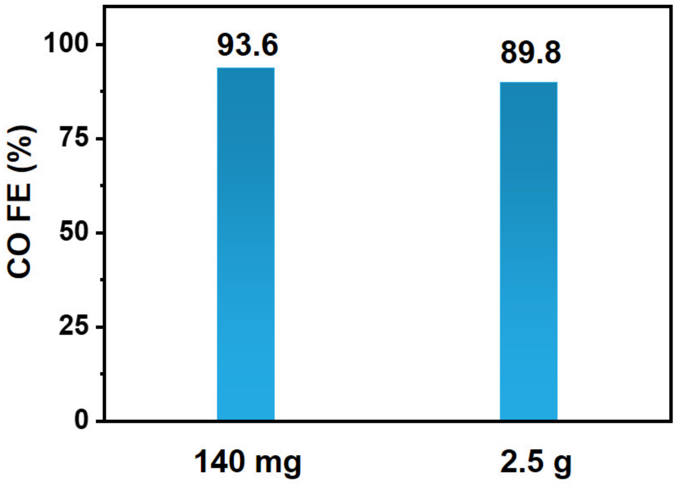


**Supplementary Figure S36.** $FE_{CO}$ obtained on Co-CNTs-MW synthesized at different scale of 140 mg and 2.5 g. The samples were biased at -0.75 V (*vs.* RHE) in the H-type cell in $CO_2$ saturated 0.5 M $KHCO_3$. The gram-scale synthesized sample exhibits no obvious decay in CO selectivity.

**Supplementary Table S1.** EXAFS fitting parameters at the Co K-edge of Co-CNTs-MW and Co-CNTs-MW after $CO_2RR$

| Sample | Path | N | R(Å) | $\sigma^2(10^{-3}\ Å^2)$ | $\Delta E_0$(eV) | R-factor |
|---|---|---|---|---|---|---|
| Co-CNTs-MW | Co-N | 3.0 | 1.92 | 3.7 | -9.6 | 0.01 |
| | Co-O | 2.1 | 2.12 | 4.0 | 1.8 | |
| Co-CNTs-MW after $CO_2RR$ | Co-N | 3.3 | 1.88 | 3.0 | -8.5 | 0.003 |
| | Co-O | 0.9 | 2.11 | 3.9 | 7.8 | |

**Supplementary Table S2.** Performance comparison of selectivity, activity and TOF for state-of-the-art $CO_2$RR electrocatalysts evaluated in H-type cell.

| Catalysts | $FE_{CO}$ | E (V vs. RHE) | $J_{CO}$ (mA $cm^{-2}$) | TOF ($h^{-1}$) | References |
|---|---|---|---|---|---|
| | 90.5% | -0.60 | 4.7 | 2871 | |
| **Co-CNTs-MW** | 93.1% | -0.80 | 18.3 | 11177 | This work |
| | 91.6% | -1.00 | 42.0 | 25896 | |
| **A-Ni-NSG**[1] | 94.0% | $-0.72^{a}$ | 22.1 | 2960 | *Nat. Energy 3*, 140–147 (2018) |
| **COF-367-Co**[2] | 91.0% | -0.67 | 3.3 | 1908 | *Science 349*, 1208 (2015) |
| **$Co$-$N_5$**[3] | 99.0% | $-0.79^{a}$ | 10.2 | 480.2 | *J. Am. Chem. Soc. 140*, 4218–4221 (2018) |
| **CoPc-CN/CNT**[4] | 98.0% | $-0.97^{a}$ | 15.0 | 14760 | *Nat. Commun. 8*, 14675 (2017) |
| **Co-TTCOF**[5] | 91.3% | -0.70 | 1.84 | 4608 | *J. Am. Chem. Soc. 139*, 8078–8081 (2017) |
| **Pd nanoparticles**[6] | 91.0% | $-0.89^{a}$ | 9.8 | 576 | *J. Am. Chem. Soc. 137*, 4288–4291 (2015) |
| **Perfluorinated CoPc**[7] | 93.0% | -0.80 | 4.4 | 5760 | *ACS Catal. 6*, 3092–3095 (2016) |
| **CATPyr/CNT**[8] | 93.0% | $-0.59^{a}$ | 0.24 | 144 | *J. Am. Chem. Soc. 138*, 2492–2495 (2016) |
| **Ni-N-C**[9] | 85.0% | $-0.83^{a}$ | 12.9 | 544 | *Nat. Commun. 8*, 944 (2017) |
| **Au nanoneedles**[10] | 95.0% | -0.35 | 22.0 | 144 | *Nature 537*, 382–386 (2016) |
| **NC-CNTs (Ni)**[11] | 90% | -1.00 | 9.3 | 11648 | *Adv. Energy Mater. 10,* 1903068 (2019) |
| **$Ni^{1}$-NCNT@$Ni_9Cu$**[12] | 97% | $-0.62^{a}$ | 32.9 | 1962 | *Angew. Chem. Int. Ed. 59*, 12055–12061 (2020) |
| **Ni SAs**[13] | 90% | -1.00 | 10.0 | 6487 | *Angew. Chem. Int. Ed. 59*, 18572–18577 (2020) |
| **$Ni_1$-N-C**[14] | 96.8% | -0.80 | 27.0 | 11315 | *Angew. Chem. Int. Ed. 59*, 20589–20595 (2020) |
| **$Fe_1NC/S_X$-Y**[15] | 96.0% | -0.50 | 6.4 | 2225 | *Adv. Mater. 32*, 2002430 (2020) |
| **$Sn$-$C_2O_2$**[16] | 80% | $-0.96^{a}$ | 16.0 | 1950 | *ACS Catal. 11*, 5212–5221 (2021) |

Notes: a means the potential was *iR*-corrected.

**Supplementary Table S3.** Full-cell energy efficiency comparison for $CO_2$-to-CO conversion of state-of-art $CO_2$RR electrocatalysts.

| Catalysts | System | Electrolyte | $FE_{CO}$ | Cell voltage (V) | *J* (mA cm$^{-2}$) | FCEE | References |
|---|---|---|---|---|---|---|---|
| **Co-CNTs-MW** | MEA | 1.0 M KOH | 98.3% | -1.90 | 50 | 68.8% | This work |
| | | | 97.5% | -2.05 | 100 | 63.3% | |
| | | | 95.4% | -2.34 | 200 | 54.1% | |
| **CoPc[17]** | MEA | 1.0 M KOH | 88.0% | -2.52 | 200 | 46.0% | *Science 365*, 367 (2019) |
| **MWNT/ PyPBI/Au[18]** | MEA | 2.0 M KOH | 85.0% | -2.25 | 158 | 49.4% | *ACS Energy Lett. 3*, 193–198 (2017) |
| **Ag/C[19]** | MEA | 1.0 M KOH | 83.0% | -2.75 | 120 | 40.1% | *ACS Energy Lett. 4*, 1770–1777 (2019) |
| **CoTMAPc[20]** | Flow cell | 1.0 M KOH | 95.6% | -2.30[a] | 239 | 55.2% | *Energy Environ. Sci. 14*, 483–492 (2021) |
| **Ag[21]** | MEA | 2.0 M KOH | / | / | 515 | 45.0% | *ACS Energy Lett,* 6, 2427–2433 (2021) |
| **Ag nanoparticles[22]** | MEA | 1.0 M KOH+0.33M Urea | 98.0% | -2.16 | 100 | 60.3% | *Angew. Chem. Int. Ed.* 60, 10577–10582 (2021) |
| **$Zn_2P_2O_7$[23]** | MEA | 1.0M KOH | 93.9% | / | 100 | 58.0% | *Angew. Chem. Int. Ed.* 61, e202202298 (2022) |
| **$Au_{24}$[24]** | MEA | 0.1M KOH | 90.0% | ~3.0 | 100 | 39.9% | *J. Am. Chem. Soc.* 144, 20, 9000–9006 (2022) |

Notes: [a] means the cell voltage was *iR*-corrected.

**Supplementary Table S4.** Faradaic efficiency of the gas products of $CO_2$RR in MEA.

| Current density (mA $cm^{-2}$) | $FE_{CO}$ (%) | $FE_{H2}$ (%) |
|---|---|---|
| 25 | 97.7±1.1 | 3.2±0.8 |
| 50 | 98.3±0.6 | 2.6±0.4 |
| 75 | 98±0.4 | 2.8±0.6 |
| 100 | 97.5±0.6 | 3.4±0.9 |
| 125 | 96.5±0.9 | 4.9±0.8 |
| 150 | 96±1.2 | 4.8±1.4 |
| 175 | 97.2±1.1 | 4.1±1.1 |
| 200 | 95.4±1.6 | 6.2±1.3 |

Notes: The Faradaic efficiency of $H_2$ and CO is about 100, which also indicates that there is no liquid phase product.

**Supplementary Table S5.** Comparison of voltage at both ends of 100 $cm^2$ MEA tested by multimeter and power output voltage under different currents.

| Current (A) | Power output (V) | Multimeter test (V) | ΔU (V) | ΔU/I (Ω) |
|---|---|---|---|---|
| 1 | 2.78 | 2.67 | 0.11 | 0.11 |
| 3 | 3.46 | 3.12 | 0.34 | 0.11 |
| 5 | 3.95 | 3.42 | 0.53 | 0.11 |
| 8 | 4.55 | 3.72 | 0.83 | 0.10 |
| 10 | 4.96 | 3.84 | 1.12 | 0.11 |

Notes: There is a resistance of 0.11 Ω in the external circuit of MEA (including wire and internal resistance of power supply).

**Supplementary Table S6.** Single-pass conversion comparison of the state-of-art $CO_2$RR electrocatalysts.

| Catalyst | Products | $I_{CO}$ (A) | Flow rate (sccm) | Electrolyte pH | SPC (%) | References |
|---|---|---|---|---|---|---|
| **Co-CNTs-MW** | CO | 4.54 | 80 | ~7 | 39.6 | This work |
| | | 8.68 | 150 | ~7 | 40.4 | |
| **Ni-SACs[25]** | CO | 8.20 | 500 | ~7 | 11.4 | *Joule* 3, 265–278 (2019) |
| **Ag[26]** | CO | 0.71 | 10.5 | ~7 | 47.0 | *Nat. Catal.* 1, 32–39 (2018) |
| **Ag[27]** | CO | 0.42 | 100 | ~7 | 3.0 | *ACS Energy Lett.* 3, 149–154 (2018) |
| **MWNT/PyPBI/ Au[18]** | CO | 0.20 | 17 | 14.3 | 8.2 | *ACS Energy Lett.* 3, 193–198 (2017) |
| **Ag[28]** | CO | 0.95 | 20 | ~7 | 33.1 | *Energy Technol.* 5, 929–936 (2017) |
| **Ag[19]** | CO | 8 | 250 | ~14 | ~22 | *ACS Energy Lett.* 4, 1770–1777 (2019) |
| **Cu-Ag[29]** | CO, $CH_3COOH$, $C_2H_4$ | 0.28 | 7 | 14 | 10.4 | *J. Am. Chem. Soc.* 140, 17, 5791–5797 (2018) |
| **Cu[30]** | $C_2H_4$ | 0.47 | 50 | 14.8 | 2.2 | *Science* 360, 783–787 (2018) |
| **Cu-PTFE[31]** | $C_2H_4$ | 1.3 | 50 | ~15 | 4.5 | *Science* 367, 661–666 (2020) |
| **Cu-PTFE[32]** | CO, HCOOH, $C_{2+}$ | 1.2 | 3 | ~1 | 77.4 | *Science* 372, 1074–1078 (2021) |

**Supplementary Table S7**. Performance comparison of the time taken to produce 1 mmol of CO per mg of catalyst and time-consuming of 1 mol CO production in the device.

| Catalyst | $FE_{CO}$ | $j_{CO}$ (mA $cm^{-2}$) | Loading amount (mg $cm^{-2}$) | Time-consuming of CO production (h $mmol^{-1}$ $mg^{-1}$) | Device reaction area ($cm^2$) | Time-consuming of 1mol CO production (h) | References |
|---|---|---|---|---|---|---|---|
| Co-CNTs-MW | 96.0% | 336.0 | 1.0 | 0.16 | 0.5 | 319.06 | This work |
| | 86.8% | 86.8 | 1.0 | 0.62 | 100.0 | 6.20 | |
| $Fe\text{-}N_4$ [33] | 90% | 33.0 | 1.0 | 1.62 | 1.0 | 1624.31 | *Adv. Mater. 33*, 2003238 (2020) |
| $Mn\text{-}N_3$ [34] | 98.3% | 18.6 | 1.0 | 2.88 | 1.0 | 2881.83 | *Nat. Commun. 11*, 4341 (2020) |
| $Fe\text{-}N_4$ [35] | 97% | 6.8 | 1.0 | 7.89 | 1.0 | 7882.67 | *Chem 7*, 1297–1307 (2021) |
| NiSA/PCFM[36] | 88% | 308.4 | 1.0 | 0.17 | 1.0 | 173.81 | *Nat. Commun. 11*, 593 (2020) |
| $In_A$/NC[37] | 97.2% | 38.3 | 1.0 | 1.40 | 0.5 | 2799.07 | *J. Am. Chem. Soc. 143*, 6877–6885 (2021) |
| Ni-NCNT@$Ni_9Cu$[12] | 97% | 32.9 | 0.5 | 0.81 | 1.0 | 1629.24 | *Angew. Chem. Int. Ed. 59*, 12055–12061 (2020) |
| (Cl, N)-Mn/G[38] | 97% | 10.0 | 0.5 | 2.68 | 1.0 | 5360.21 | *Nat. Commun. 10*, 2980 (2019) |
| $Fe^{3+}$–N–C[39] | 90% | 94.0 | 2.5 | 1.43 | 1.0 | 570.24 | *Science 364*, 1091 (2019) |
| $Co\text{-}N_2$ [40] | 94% | 18.1 | 5.0 | 14.81 | 0.5 | 5922.89 | *Angew. Chem. Int. Ed. 57*, 1944–1948 (2018) |

**Supplementary Table S8.** Stability comparison in neutral electrolyte for $CO_2$-to-CO conversion of state-of-art $CO_2$RR electrocatalysts.

| Catalyst | Electrolyte | $FE_{CO}$ | Stability (h) | References |
|---|---|---|---|---|
| **Co-CNTs-MW** | 0.1 M $KHCO_3$ | 85.6% | 60.0 | This work |
| **$Fe^{3+}$–N–C**[39] | 0.5 M $KHCO_3$ (catholyte) | ~80.0% | ~28.0 | *Science* 364, 1091–1094 (2019) |
| **NiPc–OMe MDE**[41] | 1.0 M $KHCO_3$ | ~99.0% | 40.0 | *Nat. Energy* 5, 684–692 (2020) |
| **Ni–NG**[42] | 0.1 M $KHCO_3$ | 90.0% | 8.0 | *Energy Environ. Sci.* 11, 893–903 (2018) |
| **Ni-NCB**[25] | 0.1 M $KHCO_3$ | 90.0% | 6.0 | *Joule* 3, 265–278 (2019) |
| **Mg-$C_3N_4$**[43] | 1.0 M $KHCO_3$ | 90.0% | 4.2 | *Angew. Chem. Int. Ed.* 60, 25241–25245 (2021) |
| **$H_2$–$FeN_4$/C**[35] | 0.1 M $NaHCO_3$ | ~90.0% | 24.0 | *Chem* 7, 1297–1307 (2021) |
| **Ni–Zn–N–C**[44] | 0.5 M $KHCO_3$ | 95.0% | 28.0 | *Adv. Mater.* 33, 2102212 (2021) |
| **Mn–$C_3N_4$/CNT**[34] | 0.5 M $KHCO_3$ | ~90.0% | 20.0 | *Nat. Commun.* 11, 4341 (2020) |
| **CoTAPc@CNT**[20] | 0.5 M $KHCO_3$ | ~90.0% | 12.0 | *Energy Environ. Sci.* 14, 483–492 (2021) |
| **$In_A$/NC**[37] | ionic liquid/ MeCN. | ~99.0% | 24.0 | *J. Am. Chem. Soc.* 143, 6877−6885（2021） |

## Supplementary Reference


1. Yang, H.B. et al. Atomically dispersed Ni(i) as the active site for electrochemical $CO_2$ reduction. *Nat. Energy* **3**, 140–147 (2018).
2. Lin, S. et al. Covalent organic frameworks comprising cobalt porphyrins for catalytic $CO_2$ reduction in water. *Science* **349**, 1208 (2015).
3. Pan, Y. et al. Design of single-atom Co-$N_5$ catalytic site: A robust electrocatalyst for $CO_2$ reduction with nearly 100% CO selectivity and remarkable stability. *J. Am. Chem. Soc.* **140**, 4218–4221 (2018).
4. Zhang, X. et al. Highly selective and active $CO_2$ reduction electrocatalysts based on cobalt phthalocyanine/carbon nanotube hybrid structures. *Nat. Commun.* **8**, 14675 (2017).
5. Zhu, H.-J. et al. Efficient electron transmission in covalent organic framework nanosheets for highly active electrocatalytic carbon dioxide reduction. *Nat. Commun.* **11**, 497 (2020).
6. Gao, D. et al. Size-dependent electrocatalytic reduction of $CO_2$ over Pd nanoparticles. *J. Am. Chem. Soc.* **137**, 4288–4291 (2015).
7. Morlanés, N., Takanabe, K. & Rodionov, V. Simultaneous reduction of $CO_2$ and splitting of $H_2O$ by a single immobilized cobalt phthalocyanine electrocatalyst. *ACS Catal.* **6**, 3092–3095 (2016).
8. Maurin, A. & Robert, M. Noncovalent immobilization of a molecular iron-based electrocatalyst on carbon electrodes for selective, efficient $CO_2$-to-CO conversion in water. *J. Am. Chem. Soc.* **138**, 2492–2495 (2016).
9. Ju, W. et al. Understanding activity and selectivity of metal-nitrogen-doped carbon catalysts for electrochemical reduction of $CO_2$. *Nat. Commun.* **8**, 944 (2017).
10. Liu, M. et al. Enhanced electrocatalytic $CO_2$ reduction via field-induced reagent concentration. *Nature* **537**, 382–386 (2016).
11. Fan, Q. et al. Activation of Ni particles into single Ni–N atoms for efficient

electrochemical reduction of $CO_2$. *Adv. Energy Mater.* **10**, 1903068 (2019).

12. Zhang, T. et al. Atomically dispersed nickel(I) on an alloy-encapsulated nitrogen-Doped carbon nanotube array for high-performance electrochemical $CO_2$ reduction reaction. *Angew. Chem. Int. Ed.* **59**, 12055–12061 (2020).
13. Li, Z. et al. Size-dependent nickel-based electrocatalysts for selective $CO_2$ reduction. *Angew. Chem. Int. Ed.* **59**, 18572–18577 (2020).
14. Jiao, L. et al. Single-atom electrocatalysts from multivariate metal-organic frameworks for highly selective reduction of $CO_2$ at low pressures. *Angew. Chem. Int. Ed.* **59**, 20589–20595 (2020).
15. Wang, T. et al. Gas diffusion strategy for inserting atomic iron sites into graphitized carbon supports for unusually high-efficient $CO_2$ electroreduction and high-performance Zn-$CO_2$ Batteries. *Adv. Mater.* **32**, 2002430 (2020).
16. Ni, W. et al. Nonnitrogen coordination environment steering electrochemical $CO_2$-to-CO conversion over single-atom tin catalysts in a wide potential window. *ACS Catal.* **11**, 5212–5221 (2021).
17. Ren, S. et al. Molecular electrocatalysts can mediate fast, selective $CO_2$ reduction in a flow cell. *Science* **365**, 367 (2019).
18. Verma, S. et al. Insights into the low overpotential electroreduction of $CO_2$ to CO on a supported gold catalyst in an alkaline flow electrolyzer. *ACS Energy Lett.* **3**, 193–198 (2017).
19. Endrodi, B. et al. Multilayer electrolyzer stack converts carbon dioxide to gas products at high pressure with high efficiency. *ACS Energy Lett.* **4**, 1770–1777 (2019).
20. Su, J. et al. Building a stable cationic molecule/electrode interface for highly efficient and durable $CO_2$ reduction at an industrially relevant current. *Energy Environ. Sci.* **14**, 483–492 (2021).
21. Bhargava, S.S. et al. Decreasing the energy consumption of the $CO_2$ electrolysis process using a magnetic field. *ACS Energy Lett.* **6**, 2427–2433 (2021).

22. Wang, L. et al. Regulating the local charge distribution of Ni active sites for the urea oxidation reaction. *Angew. Chem. Int. Ed.* **60**, 10577–10582 (2021).
23. Zhang, X.Y. et al. In operando identification of in situ formed metalloid Zinc$^{\delta+}$ active sites for highly efficient electrocatalyzed carbon dioxide reduction. *Angew. Chem. Int. Ed.* **61**, e202202298 (2022).
24. Kulkarni, V.K. et al. N-heterocyclic carbene-stabilized hydrido $Au_{24}$ nanoclusters: synthesis, structure, and electrocatalytic reduction of $CO_2$. *J. Am. Chem. Soc.* **144**, 9000–9006 (2022).
25. Zheng, T. et al. Large-scale and highly selective $CO_2$ electrocatalytic reduction on nickel single-atom catalyst. *Joule* **3**, 265–278 (2019).
26. Haas, T., Krause, R., Weber, R., Demler, M. & Schmid, G. Technical photosynthesis involving $CO_2$ electrolysis and fermentation. *Nat. Catal.* **1**, 32–39 (2018).
27. Salvatore, D.A. et al. Electrolysis of gaseous $CO_2$ to CO in a flow cell with a bipolar membrane. *ACS Energy Lett.* **3**, 149–154 (2018).
28. Kutz, R.B. et al. Sustainion imidazolium-functionalized polymers for carbon dioxide electrolysis. *Energy Technol.* **5**, 929–936 (2017).
29. Hoang, T.T.H. et al. Nanoporous copper-silver alloys by additive-controlled electrodeposition for the selective electroreduction of $CO_2$ to ethylene and ethanol. *J. Am. Chem. Soc.* **140,** 5791–5797 (2018).
30. Dinh, C.-T. et al. $CO_2$ electroreduction to ethylene via hydroxide-mediated copper catalysis at an abrupt interface. *Science* **360**, 783–787 (2018).
31. García de Arquer, F.P. et al. $CO_2$ electrolysis to multicarbon products at activities greater than 1 A $cm^{-2}$. *Science* **367**, 661–666 (2020).
32. Huang Jianan, E. et al. $CO_2$ electrolysis to multicarbon products in strong acid. *Science* **372**, 1074–1078 (2021).
33. Ni, W. et al. Electroreduction of carbon dioxide driven by the intrinsic defects in the carbon plane of a single Fe-$N_4$ site. *Adv. Mater.* **33**, 2003238 (2021).

34. Feng, J. et al. A Mn-$N_3$ single-atom catalyst embedded in graphitic carbon nitride for efficient $CO_2$ electroreduction. *Nat. Commun.* **11**, 4341 (2020).

35. Liu, C. et al. Constructing $FeN_4$/graphitic nitrogen atomic interface for high-efficiency electrochemical $CO_2$ reduction over a broad potential window. *Chem* **7**, 1297–1307 (2021).

36. Yang, H. et al. Carbon dioxide electroreduction on single-atom nickel decorated carbon membranes with industry compatible current densities. *Nat. Commun.* **11**, 593 (2020).

37. Guo, W. et al. Atomic indium catalysts for switching $CO_2$ electroreduction products from formate to CO. *J. Am. Chem. Soc.* **143**, 6877–6885 (2021).

38. Zhang, B. et al. Manganese acting as a high-performance heterogeneous electrocatalyst in carbon dioxide reduction. *Nat Commun* **10**, 2980 (2019).

39. Gu, J., Hsu, C.-S., Bai, L., Chen Hao, M. & Hu, X. Atomically dispersed $Fe^{3+}$ sites catalyze efficient $CO_2$ electroreduction to CO. *Science* **364**, 1091–1094 (2019).

40. Wang, X. et al. Regulation of coordination number over single Co Sites: Triggering the efficient electroreduction of $CO_2$. *Angew. Chem. Int. Ed.* **57**, 1944–1948 (2018).

41. Zhang, X. et al. Molecular engineering of dispersed nickel phthalocyanines on carbon nanotubes for selective $CO_2$ reduction. *Nat. Energy* **5**, 684–692 (2020).

42. Jiang, K. et al. Isolated Ni single atoms in graphene nanosheets for high-performance $CO_2$ reduction. *Energy Environ. Sci.* **11**, 893–903 (2018).

43. Wang, Q. et al. Atomically dispersed s-Block magnesium sites for electroreduction of $CO_2$ to CO. *Angew. Chem. Int. Ed.* **60**, 25241–25245 (2021).

44. Li, Y. et al. Synergistic effect of atomically dispersed Ni-Zn pair sites for enhanced $CO_2$ electroreduction. *Adv. Mater.* **33**, 2102212 (2021).
